# Exploring a New Generation of Pyrimidine and Pyridine Derivatives as Anti-Influenza Agents Targeting the Polymerase PA–PB1 Subunits Interaction

**DOI:** 10.3390/pharmaceutics16070954

**Published:** 2024-07-18

**Authors:** Ilaria Giacchello, Annarita Cianciusi, Chiara Bertagnin, Anna Bonomini, Valeria Francesconi, Mattia Mori, Anna Carbone, Francesca Musumeci, Arianna Loregian, Silvia Schenone

**Affiliations:** 1 Department of Pharmacy, University of Genoa, Viale Benedetto XV 3, 16132 Genoa, Italy; ilaria.giacchello@alice.it (I.G.); annar.cianciusi@gmail.com (A.C.); valeria.francesconi@edu.unige.it (V.F.); silvia.schenone@unige.it (S.S.); 2 Department of Molecular Medicine, University of Padua, Via A. Gabelli 63, 35121 Padua, Italy; chiara.bertagnin@unipd.it (C.B.); anna.bonomini@studenti.unipd.it (A.B.); arianna.loregian@unipd.it (A.L.); 3 Department of Biotechnology, Chemistry and Pharmacy, University of Siena, Via Aldo Moro 2, 53100 Siena, Italy; mattia.mori@unisi.it

**Keywords:** RdRp, PA–PB1 inhibitors, antiviral agents, protein-protein interaction inhibitors, small molecules

## Abstract

The limited range of available flu treatments due to virus mutations and drug resistance have prompted the search for new therapies. RNA-dependent RNA polymerase (RdRp) is a heterotrimeric complex of three subunits, i.e., polymerase acidic protein (PA) and polymerase basic proteins 1 and 2 (PB1 and PB2). It is widely recognized as one of the most promising anti-flu targets because of its critical role in influenza infection and high amino acid conservation. In particular, the disruption of RdRp complex assembly through protein–protein interaction (PPI) inhibition has emerged as a valuable strategy for discovering a new therapy. Our group previously identified the 3-cyano-4,6-diphenyl-pyridine core as a privileged scaffold for developing PA–PB1 PPI inhibitors. Encouraged by these findings, we synthesized a small library of pyridine and pyrimidine derivatives decorated with a thio-N-(m-tolyl)acetamide side chain (compounds **2a**–**n**) or several amino acid groups (compounds **3a**–**n**) at the C2 position. Interestingly, derivative **2d**, characterized by a pyrimidine core and a phenyl and 4-chloro phenyl ring at the C4 and C6 positions, respectively, showed an IC_50_ value of 90.1 μM in PA–PB1 ELISA, an EC_50_ value of 2.8 μM in PRA, and a favorable cytotoxic profile, emerging as a significant breakthrough in the pursuit of new PPI inhibitors. A molecular modeling study was also completed as part of this project, allowing us to clarify the biological profile of these compounds.

## 1. Introduction

Influenza, or flu, is an acute respiratory illness caused by the infection of viruses that belong to the *Orthomyxoviridae* family and are characterized by a single-stranded (ss) negative-sense (-) and segmented RNA genome. Influenza viruses are classified into A, B, C, and D types according to the antigenicity of their nucleoprotein (NP) and matrix protein 1 (M1) [1].

Influenza A viruses (IAVs) and B viruses (IBVs) are responsible for one of the most predominant infections that affect the human population, leading to widely spread seasonal flu. These viruses can cause serious illnesses, particularly among populations at high risk for influenza-associated complications. These include individuals with compromised immune systems, chronic diseases, and children and the elderly. In such cases, outcomes could include hospitalization and death [2,3].

Furthermore, various subtypes of IAV can also play a pivotal role in causing recurrent pandemics. Influenza viruses undergo persistent antigenic drifts and sporadic antigenic shifts, leading to both subtle and substantial alterations in the viral surface glycoproteins. These adaptations enable them to circumvent the pre-existing host immune response [4,5] and can also cause pandemics, such as the Spanish flu in 1918 and the most recent swine H1N1 flu [6].

The current therapeutic armamentarium against influenza virus infections is limited and poorly effective: in fact, influenza vaccines need to be constantly reformulated and do not provide protection against new potential pandemic strains [7,8]. Moreover, the currently available chemotherapeutics are few, and some of them suffer from severe side-effects and low efficacy due to the increasing chemoresistance. Among these, M2-blockers, such as amantadine and rimantadine, inhibit virus replication by blocking the M2 proton channel, and neuraminidase inhibitors, such as zanamivir and oseltamivir, prevent virus release from infected cells [9,10,11].

RNA-dependent RNA polymerase (RdRp) is widely recognized as one of the most promising anti-flu targets because of its critical role in IAV and IBV infection and high amino acid conservation. RdRp plays a key role in the viral life cycle, as it is strictly required for transcription and replication. Interestingly, this enzyme can allow virus replication only if correctly assembled [12]. The viral polymerase is a heterotrimeric complex of approximately 250 kDa, composed of polymerase acidic protein (PA) and polymerase basic proteins 1 and 2 (PB1 and PB2, respectively). The enzymatic activity of IAV and IBV RdRp entails the cleavage of the 5′-methyl cap from the host pre-mRNA. This cleaved cap structure subsequently assumes the role of a primer, triggering the synthesis of viral mRNA through a process known as “cap-snatching” [13,14]. Notably, the polymerase complex governs mRNA production and catalyzes genome replication, generating a positive-sense complementary RNA (cRNA), an intermediary molecule in replication that then functions as a template for additional copies of viral genomic segments (vRNA). The role of the three RdRp subunits has been elucidated: PB1 possesses the RNA-dependent RNA polymerase activity, is responsible for the addition of a poly(A) tail to viral mRNA, and interacts with both PA and PB2, allowing for polymerase complex assembly; PB2 binds to the 5′-methyl cap of host pre-mRNA molecules; and PA, which exhibits endonuclease activity, cleaves the pre-mRNA to produce a capped primer that is used to start transcription [1]. The search for new antiviral targets has focused on inhibiting the PA endonuclease active site, the PB2 cap-binding domain, and the catalytic activity of the PB1 subunit [6,15]. Nevertheless, a very attractive strategy is represented by the disruption of RdRp complex assembly through protein–protein interaction (PPI) inhibition [16,17,18,19]. In particular, X-ray crystallography has highlighted the potential druggability of the PA–PB1 interaction interface, which is characterized by a pocket formed by residues that are highly conserved among different virus strains. Thus, PA–PB1 PPI inhibitors could be effective against numerous viral subtypes and less susceptible to developing drug resistance. Furthermore, the specific PA–PB1 interface allows the development of small-molecule inhibitors, whose synthesis and tuning of the pharmacokinetic profile is generally easier [17,20,21,22].

**Figure 1:**
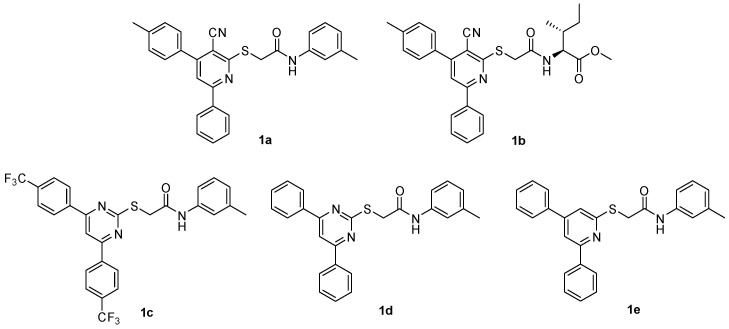
Structures of pyridine and pyrimidine compounds **1a**–**e** [23,24,25].

Using structure–activity relationship (SAR) and enzyme-linked immunosorbent assay (ELISA) evaluations, our research group previously identified the 3-cyano-4,6-diphenyl-pyridine core as a privileged scaffold for developing PA–PB1 PPI inhibitors [23,24,25]. In Figure 1, the structures of the most significant compounds, namely **1a–e**, are reported.

**Figure 2:**
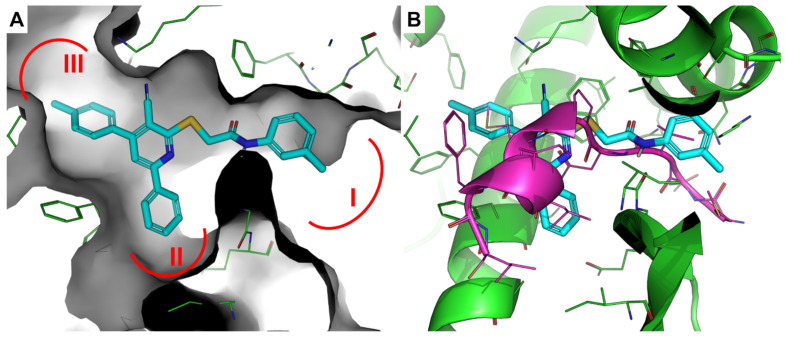
Predicted binding mode of the reference **1a** on PA at the PA–PB1 interface. (**A**) Docking pose of **1a** on PA, which is represented as a grey surface. Shape complementarity between **1a** and PA is satisfactory; hydrophobic regions, as described in [24], are highlighted in red. (**B**) Structural superimposition of the docking pose of **1a** on the crystallographic pose of PB1_N_, which is shown as magenta cartoon and lines.

In particular, the overlap between the docking pose of **1a**, i.e., one of our first synthesized cyano-pyridine derivatives (ELISA PA–PB1 IC_50_ = 80 μM) [25], and the X-ray crystallographic pose of PB1_N_ in complex with PA as described in the X-ray crystallographic structure by He et al. (PDB: 3CM8) [25,26] revealed that the cyano-pyridine core fits well into the central region naturally occupied by PB1_N_. Specifically, the carbonyl and NH of the amide portion and the cyano groups are involved in the formation of three hydrogen bonds with Gln408, Val621, and Lys643, respectively, while the aromatic rings accommodate in three sub-pockets within the PB1_N_ binding site of PA that are indicated as I, II, and III [24], which corroborates the potential competitive effect of **1a** with respect to PB1_N_, as well as its PA–PB1 dissociative effect. However, some crucial interactions made by the three N-terminal amino acids (Met1-Asp2-Val3) of the PB1_N_ peptide with PA are not established by the synthesized ligand (Figure 2) [24,25], suggesting a way for improvement of this series of PA–PB1 PPI inhibitors.

To enhance the compound’s ability to effectively displace PB1 and increase the PA binding affinity, novel hybrid compounds were designed and synthesized. These hybrids integrated the cyano-pyridine core with a peptidic or L-amino acidic chain at the C2 position. Interestingly, this substitution improved the on-target activity. Most compounds exhibited inhibition of the PPI in the micromolar range and were devoid of cytotoxicity at high concentrations in the two tested cell lines (i.e., MDCK and HEK 293T cells). Notably, among the small library of synthesized compounds, the isoleucine derivative (compound **1b**) displayed the best bioactivity profile. In fact, the compound showed inhibitory activity in the micromolar range (IC_50_ = 36 μM) in an ELISA-based PA–PB1 interaction assay, and antiviral activity, determined by plaque reduction assay (PRA) in MDCK cells, with an EC_50_ of 39 μM. The CC_50_ in MDCK and HEK 293T cells were >250 and 229 μM, respectively [23].

Furthermore, to expand our SAR knowledge, we decided to remove the cyano group from the C3 position of the heterocyclic scaffold, and either include the nitrogen atom in the ring or completely remove it, synthesizing two representative pyrimidine derivatives **1c**,**d** and a pyridine derivative **1e** (Figure 1) [24]. The obtained biological results were encouraging: all three compounds **1c**–**e** showed valuable antiviral activity, with EC_50_ values determined by PRA in MDCK cells of 26.5, 3.5, and 7.3 µM, respectively. Nevertheless, these derivatives demonstrated different toxicity and PA–PB1 inhibition capacity. Compound **1c** showed a good cytotoxicity profile (CC_50_ > 250 µM in HEK 293T and MDCK cells), although the IC_50_ value was higher than 200 µM; on the other hand, **1d** was shown to be a moderate inhibitor of PA–PB1 interaction (IC_50_ = 165 µM), but with an unsafe cytotoxicity profile (CC_50_ of 22.3 and 10.1 µM in HEK 293T and MDCK cells, respectively). Finally, the pyridine derivative **1e** showed good action as an inhibitor of the PA–PB1 interaction (IC_50_ = 52.6 µM), while being non-cytotoxic (CC_50_ > 250 µM) [24].

Encouraged by these findings, we decided to obtain a deeper insight into this family of compounds by synthesizing a small library of pyridine and pyrimidine derivatives decorated with a thio-*N*-(*m*-tolyl)acetamide side chain (compounds **2a**–**n**, Table 1) or with several amino acid groups (compounds **3a**–**n**) at the C2 position (Table 1) and different aryl groups at C4 and C6 positions to explore the two hydrophobic areas II and III of the PA subunit. In both series, we maintained the thio-alkyl linker, since thioacetamide derivatives of previous synthesis (compounds **1a**–**e**) showed significant activity in in vitro assays.

We carefully chose the compounds to synthesize on the basis of a specific rationale for each subgroup of molecules. For compounds **2a**–**n**, we diversified the aromatic rings to explore the chemical space around the heterocyclic core. In particular, for compounds **2f**–**h**, we selected the naphthalene ring to investigate the effect of a bulkier planar cycle. For compounds **3a**–**n**, we selected the amino acids that yielded the most active compounds in previous studies [23].

Herein, we report the synthesis and the biological evaluation of the new library of compounds. Furthermore, the study involved the conduction of molecular modeling simulations to provide structural hints that further elucidate the activity profile of representative compounds.

## 2. Materials and Methods

### 2.1. Chemistry

All commercially available chemicals were used as purchased. DCM was dried over calcium hydride. Anhydrous reactions were run under a positive pressure of dry N_2_ or argon. TLC was carried out using Merck TLC plates silica gel 60 F254. Chromatographic purifications were performed on columns packed with Merk 60 silica gel, 23–400 mesh, for the flash technique. ^1^H NMR and ^13^C NMR spectra were recorded on a Brucker Avance DPX400 (at 400 MHz for ^1^H and 101 MHz for ^13^C) or using a Varian Gemini 200 (200 MHz for ^1^H) in a DMSO-*d*_6_ or CDCl_3_ solvent as indicated. Chemical shifts (δ) were expressed in parts per million (ppm) relative to tetramethylsilane (TMS), which was used as the internal standard. Data are shown as follows: chemical shift, multiplicity (s = singlet, d = doublet, t = triplet, q = quartet, quint = quintet, sx = sextet, dd = doublet of doublets, tt = triple of triplets, bs = broad singlet), coupling constant (*J*) in Hertz (Hz), and integration. Elemental analysis for C, H, N, and S was determined using Thermo Scientific Flash 2000, and results were within ±0.4% of the theoretical value. All target compounds possessed a purity of ≥95%, verified by elemental analysis.

#### 2.1.1. General Procedure for the Synthesis of Compounds **5a**,**b**

To a solution of 2,4,6-trichloropyrimidine **4a** (1.00 g, 5.45 mmol) in THF (9 mL), an excess of the opportune phenylboronic acid (10.90 mmol), Pd(OAc)_2_ (24.47 mg, 0.11 mmol), PPh_3_ (42.88 mg, 0.16 mmol), and 1 M Na_2_CO_3_ (9 mL) were added. The resulting mixture was stirred at reflux under a nitrogen atmosphere for 3 h. Then, the solvent was removed under vacuum and the reaction was extracted with CH_2_Cl_2_ (3 × 40 mL). The organic phase was washed with water (100 mL), dried over Na_2_SO_4_, filtered, and concentrated under reduced pressure. The crude product was purified by column chromatography using PE (bp 40–60 °C)/CH_2_Cl_2_ (6:4) (**5a**) or (1:1) (**5b**) as the eluent to produce the compounds **5a**,**b** as white solids.

*2-Chloro-4,6-bis(4-chlorophenyl)pyrimidine (***5a***)*. Yield: 61%. ^1^H NMR (200 MHz, CDCl_3_): δ (ppm) 7.52–7.56 (m, 4H, Ar), 7.99 (s, 1H, pyr), 8.04–8.15 (m, 4H, Ar). Anal. calcd. for C_16_H_9_Cl_3_N_2_: C 57.26, H 2.70, N 8.35; found C 57.19, H 2.64, N 8.20.
*2-Chloro-4,6-bis(4-fluorophenyl)pyrimidine (***5b***)*. Yield: 47%. ^1^H NMR (200 MHz, CDCl_3_): δ (ppm) 7.21–7.28 (m, 4H, Ar), 7.95 (s, 1H, pyr), 8.15–8.22 (m, 4H, Ar). Anal. calcd. for C_16_H_9_ClF_2_N_2_: C 63.49, H 3.00, N 9.25; found C 63.47, H 3.28, N 9.44.

#### 2.1.2. General Procedure for the Synthesis of 2,4-Dichloro-6-phenylpyrimidines **6a**–**c**

To a solution of 2,4,6-trichloropyrimidine (1.00 g, 5.45 mmol) **4a** in THF (9 mL), the appropriate phenylboronic acid (4.52 mmol), Pd(OAc)_2_ (24.47 mg, 0.11 mmol), PPh_3_ (57.70 mg, 0.22 mmol), and 1 M Na_2_CO_3_ (9 mL) were added. The resulting mixture was stirred at reflux under nitrogen atmosphere for 3–6 h. Then, the solvent was removed under vacuum and the reaction extracted with CH_2_Cl_2_ (3 × 30 mL). The organic phase was washed with water (90 mL), dried over Na_2_SO_4_, filtered, and concentrated under reduced pressure. The crude product was purified by column chromatography using PE (bp 40–60 °C)/AcOEt (95:5) as the eluent to afford the compounds **6a**–**c** as white solids.

*2,4-Dichloro-6-phenylpyrimidine (***6a***)* [27]. Yield: 90%. ^1^H NMR (200 MHz, CDCl_3_): δ (ppm) 7.55–7.61 (m, 3H, Ar), 7.71 (s, 1H, pyr), 8.07–8.13 (m, 2H, Ar). Anal. calcd. for C_10_H_6_Cl_2_N_2_: C 53.37, H 2.69, N 12.45; found C 53.65, 2.58, N 12.37.
*2,4-Dichloro-6-(4-chlorophenyl)pyrimidine (***6b***)*. Yield: 83%. ^1^H NMR (200 MHz, CDCl_3_): δ (ppm) 7.53 (d, *J* = 8.0 Hz, 2H, Ar), 7.68 (s, 1H, pyr), 8.06 (d, *J* = 8.0 Hz, 2H, Ar). Anal. calcd. for C_10_H_5_Cl_3_N_2_: C 46.28, H 1.94, N 10.79; found C 46.47, H 2.14, N 10.51.
*2,4-Dichloro-6-(4-fluorophenyl)pyrimidine (***6c***)*. Yield: 35%. ^1^H NMR (200 MHz, CDCl_3_): δ (ppm) 7.20–7.29 (m, 2H, Ar), 7.67 (s, 1H, pyr), 8.10–8.17 (m, 2H, Ar). Anal. calcd. for C_10_H_5_Cl_2_FN_2_: C 49.42, H 2.07, N 11.53; found C 49.59, H 1.96, N 11.63.

#### 2.1.3. Synthesis of 2-Chloro-6-phenylpyrimidine-4-carboxylic Acid **6d**

To a solution of 2,6-dichloropyrimidine-4-carboxylic acid **4b** (0.50 g, 2.59 mmol) in THF (5 mL), phenylboronic acid (0.26 g, 2.15 mmol), Pd(OAc)_2_ (11.63 mg, 0.05 mmol), PPh_3_ (20.38 mg, 0.08 mmol), and 1 M Na_2_CO_3_ (4.50 mL) were added. The resulting mixture was stirred at reflux under nitrogen atmosphere for 6 h. Then, the solvent was removed under vacuum, 1 N HCl was added, and the reaction extracted with CH_2_Cl_2_ (3 × 30 mL). The organic phase was washed with water (90 mL), dried over Na_2_SO_4_, filtered, and concentrated under reduced pressure. The crude residue was used in the next reaction without any further purification.

#### 2.1.4. General Procedure for the Synthesis of Compounds **5c**–**e**

To a solution of intermediates **6a**,**b** (1.00 g, 5.45 mmol) in THF (9 mL), the opportune phenylboronic acid (5.45 mmol), Pd(OAc)_2_ (24.47 mg, 0.11 mmol), PPh_3_ (42.88 mg, 0.16 mmol), and 1 M Na_2_CO_3_ (9 mL) were added. The resulting mixture was stirred at reflux under nitrogen atmosphere for 3 h. Then, the solvent was removed under vacuum and the reaction extracted with CH_2_Cl_2_ (3 × 30 mL). The organic phase was washed with water (90 mL), dried over Na_2_SO_4_, filtered, and concentrated under reduced pressure. The crude product was purified by column chromatography using PE (bp 40–60 °C)/AcOEt (95:5) (**5c**), *n*-hexane/AcOEt (95:5) (**5d**), or (98:2) (**5e**) as the eluent to afford the compounds **5c**–**e** as white solids.

*2-Chloro-4-(4-chlorophenyl)-6-phenylpyrimidine (***5c***)*. Yield: 45%. ^1^H NMR (200 MHz, CDCl_3_): δ (ppm) 7.57–7.60 (m, 5H, Ar), 8.02 (s, 1H, pyr), 8.15–8.19 (m, 4H, Ar). Anal. calcd. for C_16_H_10_Cl_2_N_2_: C 63.81, H 3.35, N 9.30; found C 63.65, H 3.37, N 9.31.
*2-Chloro-4-(4-fluorophenyl)-6-phenylpyrimidine (***5d***)*. Yield: 63%. ^1^H NMR (200 MHz, CDCl_3_): δ (ppm) 7.24–7.30 and 7.56–7.60 (2m, 5H, Ar), 8.00 (s, 1H, pyr), 8.15–8.21 (m, 4H, Ar). Anal. calcd. for C_16_H_10_ClFN_2_: C 67.50, H 3.54, N 9.84; found C 67.44, H 3.84, N 9.89.
*2-Chloro-4-(4-chlorophenyl)-6-(4-fluorophenyl)pyrimidine (***5e***)*. Yield: 24%. ^1^H NMR (200 MHz, CDCl_3_): δ (ppm) 7.26–7.30 and 7.52–7.57 (2m, 4H, Ar), 7.97 (s, 1H, pyr), 8.11–8.21 (m, 4H, Ar). Anal. calcd. for C_16_H_9_Cl_2_FN_2_: C 60.21, H 2.84, N 8.78; found C 60.09, H 2.78, N 8.98.

#### 2.1.5. General Procedure for the Synthesis of 2-Chloro-4-(naphthalen-1-yl)-6-phenylpyrimidines **5f**–**h**

To a solution of the opportune intermediates **6a**–**c** (2.00 mmol) in DME (10 mL), naphthalene boronic acid (0.38 g, 2.20 mmol), Pd(OAc)_2_ (22.45 mg, 0.10 mmol), PPh_3_ (52.36 mg, 0.20 mmol), and Na_2_CO_3_ (0.66 g, 6.20 mmol) dissolved in the minimum amount of water_,_ were added. The resulting mixture was stirred at reflux under nitrogen atmosphere for 3 h. Then, solvent was removed under vacuum and the reaction extracted with CH_2_Cl_2_ (3 × 30 mL). The organic phase was washed with water (90 mL), dried over Na_2_SO_4_, filtered, and concentrated under reduced pressure. The crude product was purified by column chromatography using PE (bp 40–60 °C)/Et_2_O (8:2) (**5f**), CH_2_Cl_2_ (**5g**), or *n*-hexane/AcOEt (9:1) (**5h**) as the eluent to afford the compounds **5f**–**h** as white solids.

*2-Chloro-4-(1-naphthyl)-6-phenylpyrimidine (***5f***)*. Yield: 43%. ^1^H NMR (200 MHz, CDCl_3_): δ (ppm) 7.58–7.65 (m, 6H, Ar), 7.75 (s, 1H, pyr), 7.96–7.98 and 8.00–8.20 (2m, 6H, Ar). Anal. calcd. for C_20_H_13_ClN_2_: C 75.83, H 4.14, N 8.84; found C 75.93, H 4.21, N 8.69.
*2-Chloro-4-(4-chlorophenyl)-6-(1-naphthyl)pyrimidine (***5g***)*. Yield: 36%. ^1^H NMR (200 MHz, CDCl_3_): δ (ppm) 7.52–7.63 (m, 5H, Ar), 7.94 (s, 1H, pyr), 7.96–8.17 (m, 6H, Ar). Anal. calcd. for C_20_H_12_Cl_2_N_2_: C 68.39, H 3.44, N 7.98; found C 68.30, H 3.31, N 8.33.
2-Chloro-4-(4-fluorophenyl)-6-(1-naphthyl)pyrimidine (**5h***)*. Yield: 73%. ^1^H NMR (200 MHz, CDCl_3_): δ (ppm) 7.21–7.25, 7.58–7.66 and 7.75–7.79 (3 m, 7H, Ar) 7.92 (s, 1H, pyr), 7.93–8.06 and 8.18–8.25 (2m, 4H, Ar). Anal. calcd. for C_20_H_12_ClFN_2_: C 71.75, H 3.61, N 8.37; found C 71.63, H 3.46, N 8.30.

#### 2.1.6. Synthesis of 2-Chloro-4-phenyl-6-(phenylthio)pyrimidine **5i**

To a solution of NaOH (0.43 g, 1.07 mmol) in water (7 mL) and acetone (7 mL), thiophenol (0.14 mL, 1.33 mmol) was added; the mixture was stirred at room temperature for 1 h. Then, **6a** (0.30 g, 1.33 mmol) was added. The mixture was stirred at room temperature, and after 2 h, NaOH pellets (0.43 g, 1.07 mmol) were added. The reaction was stirred at room temperature for an additional 1 h. Then, acetone was removed under vacuum and the reaction extracted with AcOEt (3 × 20 mL). The organic phase was washed with water (60 mL), dried over Na_2_SO_4_, filtered, and concentrated under reduced pressure. The crude product was purified by column chromatography using PE (bp 40–60 °C)/CH_2_Cl_2_ (6:4) as the eluent to give the desired compound **5i** as a white solid. Yield: 71%. ^1^H NMR (200 MHz, CDCl_3_): δ (ppm) 7.01 (s, 1H, pyr), 7.44–7.71 and 7.82–7.86 (2m, 10H, Ar). Anal. calcd. for C_16_H_11_ClN_2_S: C 64.32, H 3.71, N 9.38, S 10.73; found C 64.44, H 3.80, N 9.68, S 10.53.

#### 2.1.7. General Procedure for the Synthesis of 2-Chloro-4-(4-substitued phenyl)-6-(phenylthio)pyrimidines **5j**,**k**

To a suspension of NaH (60% dispersion in mineral oil, 0.16 g, 4.00 mmol) in anhydrous DMF (9 mL) at 0 °C, thiophenol (0.10 mL, 1.00 mmol) was added. The mixture was stirred at 0 °C under nitrogen atmosphere for 1 h, then a solution of the opportune derivatives **6b**,**c** (1.00 mmol) in anhydrous DMF (2 mL) was added. The reaction was stirred at room temperature under nitrogen atmosphere for 3 h. Then, water was added dropwise under cooling in an ice bath, and the reaction extracted with CH_2_Cl_2_ (3 × 30 mL). The organic phase was washed with water (90 mL), dried over Na_2_SO_4_, filtered, and concentrated under reduced pressure. The crude product was purified by column chromatography using *n*-hexane/AcOEt (95:5) as the eluent to afford the compounds **5j**,**k** as white solids.

*2-Chloro-4-(4-chlorophenyl)-6-(phenylthio)pyrimidine (***5j***)*. Yield: 47%. ^1^H NMR (200 MHz, CDCl_3_): δ (ppm) 6.86 (s, 1H, pyr), 7.33–7.48 and 7.57–7.69 (2m, 9H, Ar). Anal. calcd. for C_16_H_10_Cl_2_N_2_S: C 57.67, H 3.02, N 8.41, S 9.62; found C 57.55, H 3.31, N 8.76, S 9.92.
*2-Chloro-4-(4-fluorophenyl-6-(phenylthio)pyrimidine (***5k***)*. Yield: 71%. ^1^H NMR (200 MHz, CDCl_3_): δ (ppm) 6.85 (s, 1H, pyr), 7.41–7.43, 7.57–7.62 and 7.65–7.75 (3 m, 9H, Ar). Anal. calcd. for C_16_H_10_ClFN_2_S: C 60.67, H 3.18, N 8.84, S 10.12; found C 60.52, H 3.30, N 8.51, S 10.45.

#### 2.1.8. Synthesis of 2-Chloro-N,6-diphenylpyrimidine-4-carboxamide **5l**

To a solution of intermediate **6d** (0.61 g, 2.60 mmol) in anhydrous DMF (8 mL), aniline (0.28 mL, 3.13 mmol), EDC (0.60 g, 3.13 mmol), and HOBt (0.35 g, 2.60 mmol) were added. The mixture was stirred under nitrogen atmosphere at room temperature for 3 h. Then, the reaction was quenched with water and extracted with AcOEt (3 × 30 mL). The organic phase was washed with saturated NaHCO_3_ solution (2 × 30 mL) and with saturated NaCl solution (2 × 30 mL), dried over Na_2_SO_4_, filtered, and concentrated under reduced pressure. The crude product was purified by column chromatography using *n*-hexane/AcOEt (7:3) as the eluent to obtain the desired compound **5l** as an off-white solid. Yield: 20%. ^1^H NMR (200 MHz, CDCl_3_): δ (ppm) 7.24–7.27, 7.42–7.62, 7.81–7.84 and 8.23–8.28 (4 m, 10H, Ar), 8.60 (s, 1H, pyr), 9.70 (br s, 1H, NH). Anal. calcd. for C_17_H_12_ClN_3_O: C 65.92, H 3.91, N 13.57; found C 66.04, H 3.98, N 13.49.

#### 2.1.9. Synthesis of 2-Chloro-4,6-diphenylpyridine **8a**

2,6-Dichloro-4-iodopyridine **7** (0.60 g, 2.20 mmol), tetrakis(triphenylphosphine)palladium (0) (10 mol %), and phenylboronic acid (0.53 g, 4.40 mmol) were placed into a reaction flask, which had been previously purged and backfilled with nitrogen flow. Then, DME (22 mL) and an aqueous solution of 1 M K_2_CO_3_ (6.6 mL) were added. The reaction was stirred under reflux for 16 h. Then, the reaction mixture was filtered, and the solvent concentrated under reduced pressure. The crude residue was extracted with DCM (20 mL), washed with H_2_O (20 mL), and dried over Na_2_SO_4_. The solvent was evaporated under vacuum, and the crude product was purified by column chromatography using PE (bp 40–60 °C)/AcOEt (9:1) as the eluent to give the desired compound as a pale-yellow oil. Yield: 60%. ^1^H NMR (400 MHz, CDCl_3_): δ (ppm) 7.40–7.56 (m, 7H, Ar + CH-Py), 7.65 (dt, *J* = 7.8, 1.7 Hz, 2H, Ar), 7.73–7.90 (m, 1H, CH-Py), 8.04 (dt, *J* = 8.1, 1.6 Hz, 2H, Ar). Anal. calcd. for C_17_H_12_ClN: C, 76.84; H, 4.55; N, 5.27; found: C, 76.64; H, 4.62; N, 4.95.

#### 2.1.10. General Procedure for the Synthesis of 2,6-Dichloro-4-phenylpyridines **9a**,**b**

To a solution of 2,6-dichloro-4-iodopyridine **7** (0.50 g, 1.83 mmol) in THF (6 mL), the opportune phenylboronic acid (2.01 mmol), Pd(OAc)_2_ (8.20 mg, 0.04 mmol), PPh_3_ (14.36 mg, 0.05 mmol), and 1 M Na_2_CO_3_ (4.50 mL) were added. The resulting mixture was stirred at reflux under nitrogen atmosphere for 3 h. Then, the solvent was removed under vacuum, and the reaction extracted with CH_2_Cl_2_ (3 × 30 mL). The organic phase was washed with water, dried over Na_2_SO_4_, filtered, and concentrated under reduced pressure. The crude product was purified by column chromatography using PE (bp 40–60 °C)/AcOEt (95:5) as the eluent to afford the compounds **9a**,**b** as white solids.

*2,6-Dichloro-4-phenylpyridine* (**9a**). Yield: 73%. ^1^H NMR (200 MHz, CDCl_3_): δ (ppm) 7.49 (s, 2H, pyr), 7.50–7.54 and 7.60–7.63 (2m, 5H, Ar). Anal. calcd. for C_11_H_7_Cl_2_N: C 58.96, H 3.15, N 6.25; found C 58.88, H 3.12, N 5.96.
*2,6-Dichloro-4-(4-chlorophenyl)pyridine (***9b***)*. Yield: 74%. ^1^H NMR (200 MHz, CDCl_3_): δ (ppm) 7.47 (s, 2H pyr), 7.54–7.55 (m, 4H Ar). Anal. calcd. for C_11_H_6_Cl_3_N: C 51.11, H 2.34, N 5.42; found C 51.14, H 2.25, N 5.07.

#### 2.1.11. General Procedure for the Synthesis of 2-Chloro-4-phenyl-6-(phenylthio)pyridines **8b**,**c**

To a suspension of NaH (60% dispersion in mineral oil, 0.16 g, 4.00 mmol) in anhydrous DMF (9 mL) at 0 °C, thiophenol (0.10 mL, 1.00 mmol) was added. The mixture was stirred at 0 °C under nitrogen atmosphere for 1 h. Then, a solution of the opportune derivatives **9a**,**b** (1.00 mmol) in anhydrous DMF (2 mL) was added, and the reaction was stirred at room temperature under nitrogen for 3 h. Water was added dropwise under cooling in an ice bath, and the reaction was extracted with CH_2_Cl_2_ (3 × 30 mL). The organic phase was washed with water, dried over Na_2_SO_4_, filtered, and concentrated under reduced pressure. The crude product was purified by column chromatography using *n*-hexane/AcOEt (95:5) as the eluent to afford the compounds **8b**,**c** as white solids.

*2-Chloro-4-phenyl-6-(phenylthio)pyridine (***8b***)*. Yield: 95%. ^1^H NMR (200 MHz, CDCl_3_): δ (ppm) 6.94 (s, 1H, pyr), 7.25 (s, 1H, pyr), 7.44–7.51 and 7.63–7.70 (2m, 10H, Ar). Anal. calcd. for C_17_H_12_ClNS: C 68.56, H 4.06, N 4.70, S 10.77; found C 68.41, H 3.90, N 5.00, S 11.11.
*2-Chloro-4-(4-chlorophenyl)-6-(phenylthio)pyridine (***8c***)*. Yield: 82%. ^1^H NMR (200 MHz, CDCl_3_): δ (ppm) 6.88 (s, 1H, pyr), 7.21 (s, 1H, pyr), 7.37–7.52 and 7.65–7.68 (2m, 9H, Ar). Anal. calcd. for C_17_H_11_Cl_2_NS: C 61.46, H 3.34, N 4.22, S 9.65; found C 61.40, H 3.54, N 4.45, S 9.82.

#### 2.1.12. Synthesis of (E)-1-(4-Fluorophenyl)-3-phenylprop-2-en-1-one **12** [28]

A solution of benzaldehyde **10** (0.6 mL, 5.80 mmol) in EtOH (25 mL) and 4 N NaOH (3.63 mL) were added to a solution of 4-fluoro-acetophenone **11** (0.7 mL, 5.80 mmol) in EtOH (25 mL). The mixture was stirred at room temperature for 3 h. The reaction was quenched with 2 N HCl, filtered, and the solvent was partially evaporated under vacuum. The crude residue was dissolved in AcOEt, washed with H_2_O, and dried over Na_2_SO_4_. The product was purified by column chromatography using a mixture of petroleum ether and AcOEt (9:1) as the eluent, affording **12** as a yellowish solid product. Yield: 82%. ^1^H NMR (400 MHz, DMSO-*d*_6_): δ (ppm) 7.26–7.40 (m, 2H, Ar), 7.39–7.49 (m, 3H, Ar), 7.71 (d, *J* = 15.6 Hz, 1H, C=CH), 7.80–7.89 (m, 2H, *p*F-Ar), 7.92 (d, *J* = 15.6 Hz, 1H, C=CH), 8.06–8.30 (m, 2H, *p*F-Ar). Anal. calcd. for C_15_H_11_FO: C, 79.63; H, 4.90; found C, 79.45; H, 5.03.

#### 2.1.13. Synthesis of 6-(4-Fluorophenyl)-4-phenylpyridin-2(1H)-one **13**

A mixture of 1-(4-fluorophenyl)-3-phenylprop-2-en-1-one **12** (0.350 g, 1.0 mmol), ethyl 2-nitroacetate (0.17 mL; 1.0 mmol), and NH_4_OAc (0.717 g; 6.0 mmol) in EtOH (8.0 mL) was refluxed under nitrogen atmosphere for 6 h. The reaction mixture was cooled to room temperature and the solid obtained was filtered and recrystallized from EtOH to give the pure product **13**. Yield: 25%. ^1^H NMR (400 MHz, DMSO-*d*_6_): δ (ppm) 6.64 (s, 1H, CH-Py), 7.01 (s, 1H, CH-Py), 7.30 (t, *J* = 8.8 Hz, 2H, Ar), 7.39–7.59 (m, 3H, Ar), 7.66–7.84 (m, 2H, *p*F-Ar), 7.85–8.15 (m, 2H, *p*F-Ar), 11.59 (s, 1H, NH-Py), Anal. calcd. for C_17_H_12_FNO: C, 76.97; H, 4.56; N, 5.28; found: C, 76.99; H, 4.52; N, 5.30.

#### 2.1.14. Synthesis of 2-Chloro-6-(4-fluorophenyl)-4-phenylpyridine **8d**

The Vilsmeier complex, previously prepared from POCl_3_ (0.89 mL, 9.6 mmol) and anhydrous DMF (2.98 mL, 9.6 mmol), was added to a solution of **13** (0.210 g, 1.0 mmol) in CHCl_3_ (15 mL). The mixture was refluxed for 12 h. The solution was washed with H_2_O (10 mL), dried over Na_2_SO_4_, filtered, and concentrated under reduced pressure. The crude residue was purified by column chromatography using PE (bp 40–60 °C)/AcOEt (9:1) as the eluent to give the compound **8d** as a white solid. Yield: 60%. ^1^H NMR (400 MHz, DMSO-*d*_6_): δ (ppm) 7.02–7.40 (m, 2H, Ar), 7.40–7.60 (m, 3H, Ar), 7.77 (d, *J* = 1.3 Hz, 1H, CH-Py), 7.86–8.06 (m, 2H, *p*F-Ar), 8.21–8.24 (m, 3H, *p*F-Ar + CH-Py). Anal. calcd. for C_17_H_11_ClFN: C, 71.97; H, 3.91; N, 4.94; found: C, 72.00; H, 3.93; N, 4.97.

#### 2.1.15. General Procedure for the Synthesis of Compounds **2a**–**l**

To a suspension of the appropriate intermediate **5a**–**l** (1.00 mmol) in anhydrous acetonitrile at 0 °C, 2-mercapto-*N*-(*m*-tolyl)acetamide **14** (0.20 g, 1.10 mmol) and anhydrous K_2_CO_3_ (0.21 g, 1.50 mmol) were added. The reaction was stirred at room temperature under nitrogen atmosphere for 5 h. Then, the solvent was removed under vacuum and the reaction extracted with CH_2_Cl_2_ (3 × 30 mL). The organic phase was washed with water (90 mL), dried over Na_2_SO_4_, filtered, and concentrated under reduced pressure. The crude product was purified by column chromatography using CH_2_Cl_2_ as the eluent to obtain the final compounds **2a**–**l** as white solids.

*2-{[4,6-Bis(4-chlorophenyl)pyrimidin-2-yl]thio}-N-(m-tolyl)acetamide (***2a***)*. Yield: 20%. Mp: 225–227 °C. ^1^H NMR (200 MHz, CDCl_3_): δ (ppm) 2.19 (s, 3H, CH_3_), 4.03 (s, 2H, CH_2_), 6.83 (d, *J* = 8.0 Hz, 2H, Ar), 6.96 (s, 1H, Ar), 7.08 and 7.15 (2 t, *J* = 8.0 Hz, 2H, Ar), 7.52 (d, *J* = 8.4 Hz, 4H, Ar), 7.78 (s, 1H, H-5), 8.09 (d, *J* = 8.4 Hz, 4H, Ar), 8.96 (br s, 1H, NH). ^13^C NMR (101 MHz, DMSO-*d*_6_): δ (ppm) 21.68, 36.38, 108.86, 116.77, 120.08, 124.52, 129.19, 129.41, 129.87, 135.12, 138.46, 139.62, 163.80, 166.87, 171.56. Anal. calcd. for C_25_H_19_Cl_2_N_3_OS: C 62.50, H 3.99, N 8.75, S 6.67; found C 62.43, H 4.25, N 8.83, S 6.60.
*2-{[4,6-Bis(4-fluorophenyl)pyrimidin-2-yl]thio}-N-(m-tolyl)acetamide (***2b***)*. Yield: 23%. Mp: 210–211 °C. ^1^H NMR (200 MHz, CDCl_3_): δ (ppm) 2.18 (s, 3H, CH_3_), 4.04 (s, 2H, CH_2_), 6.82 (d, *J* = 7.2 Hz, 1H, Ar), 6.99 (s, 1H, Ar), 7.07 (t, *J* = 7.8 Hz, 1H, Ar), 7.13 (d, *J* = 8.0 Hz, 1H, Ar), 7.21–7.26 (m, 4H, Ar), 7.76 (s, 1H, H-5), 8.14–8.17 (m, 4H, Ar) 9.03 (br s, 1H, NH). ^13^C NMR (101 MHz, CDCl_3_): δ (ppm) 21.42, 35.96, 108.37, 116.30, 116.52, 116.71, 120.28, 125.09, 128.86, 129.60, 129.69, 129.69, 132.37, 132.40, 137.79, 138.85, 164.53, 165.07 (d, *J*_C-F_ = 263 Hz), 165.83 (d, *J*_C-F_ = 263 Hz), 170.99. Anal. calcd. for C_25_H_19_F_2_N_3_OS: C 67.10, H 4.28, N 9.39, S 7.16; found C 67.35, H 4.11, N 9.34, S 6.94.
*2-{[4-(4-Chlorophenyl)-6-phenylpyrimidin-2-yl]thio}-N-(m-tolyl)acetamide (***2c***)*. Yield: 20%. Mp: 204–206 °C. ^1^H NMR (200 MHz, CDCl_3_): δ (ppm) 2.19 (s, 3H, CH_3_), 4.09 (s, 2H, CH_2_), 6.81 (d, *J* = 7.6 Hz, 1H, Ar), 6.91 (s, 1H, Ar), 7.03–7.14 (m, 2H, Ar), 7.51–7.57 (m, 5H, Ar), 7.82 (s, 1H, H-5), 8.09–8.15 (m, 4H, Ar), 9.14 (br s, 1H, NH). ^13^C NMR (100 MHz, CDCl_3_): δ (ppm) 21.40, 35.95, 108.90, 116.68, 120.24, 124.98, 127.51, 128.80, 129.53, 131.73, 134.71, 136.25, 138.78, 164.43, 165.79, 167.27,171.19. Anal. calcd. for C_25_H_20_ClN_3_OS: C 67.33, H 4.52, N 9.42, S 7.19; found C 67.25, H 4.54, N 9.10, S 7.40.
*2-{[4-(4-Fluorophenyl)-6-phenylpyrimidin-2-yl]thio}-N-(m-tolyl)acetamide (***2d***)*. Yield: 20%. Mp: 186–190 °C. ^1^H NMR (400 MHz, CDCl_3_): 2.14 (s, 3H, CH_3_), 4.03 (s, 2H, CH_2_), 6.79 (d, *J* = 7.5 Hz, 1H, Ar), 6.88 (s, 1H, Ar), 7.02–7.12 (m, 2H, Ar), 7.19–7.25 (m, 2H, Ar), 7.54–7.56 (m, 3H, Ar), 7.80 (s, 1H, H-5), 8.11–8.17 (m, 4H, Ar), 9.18 (br s, 1H, NH). ^13^C NMR (101 MHz, CDCl_3_): δ (ppm) 21.40, 35.93, 108.80, 116.26, 116.48, 116.65, 120.22, 124.94, 127.48, 128.80, 129.35, 129.61, 129.70, 131.66, 136.30, 137.86, 138.76, 164.50, 165.66, 167.05 (d, *J*_C-F_ = 254 Hz), 167.32, 171.09. Anal. calcd. for C_25_H_20_FN_3_OS: C 69.91, H 4.69, N 9.78, S 7.46; found C 69.75, H 4.82, N 10.05, S 7.41.
*2-{[4-(4-Chlorophenyl)-6-(4-fluorophenyl)pyrimidin-2-yl]thio}-N-(m-tolyl)acetamide (***2e***)*. Yield: 23%. Mp: 213–217 °C. ^1^HNMR (400 MHz, DMSO-*d*_6_): 2.22 (s, 3H, CH_3_), 4.14 (s, 2H, CH_2_), 6.83 (d, *J* = 7.5 Hz, 1H, Ar), 7.16 (t, *J* = 7.8 Hz, 1H, Ar), 7.25 (m, 2H, Ar), 7.39 (d, *J* = 8.1 Hz, 1H, Ar), 7.42 (s, 1H, Ar), 7.48 (d, *J* = 8.6 Hz, 2H, Ar), 8.28–8.32 (m, 3H, 2H Ar + H-5), 8.34–8.38 (m, 2H, Ar), 10.29 (br s, 1H, NH). ^13^CNMR (101 MHz, DMSO-*d*_6_): δ (ppm) 21.68, 36.37, 108.66, 116.41, 116.76, 120.07, 124.50, 129.17, 129.38, 129.83, 130.65, 132.78, 135.16, 136.77, 138.45, 139.61, 163.64, 163.91, 164.71 (d, *J*_C-F_ = 248 Hz), 166.88, 171.44. Anal. Calcd. For C_25_H_19_ClFN_3_OS: C 64.72, H 4.13, N 9.06, S 6.91; found C 64.88, H 4.39, N 9.38, S 7.18.
*2-{[4-(Naphthalen-1-yl)-6-phenylpyrimidin-2-yl)thio)-N-(m-tolyl)acetamide (***2f***)*. Yield: 20%. Mp: 147–148 °C. ^1^H NMR (400 MHz, CDCl_3_): δ (ppm) 2.02 (s, 3H, CH_3_), 3.99 (s, 2H, CH_2_), 6.56 (s, 1H, Ar), 6.71–6.73 (m, 1H, Ar), 6.93–7.00 (m, 2H, Ar), 7.51–7.57 (m, 5H, Ar), 7.75 (dd, *J* = 7.1, 1.2 Hz, 1H, Ar), 7.77 (s, 1H, H-5), 7.96 (dd, *J* = 7.4, 2.1 Hz, 1H, Ar), 8.03 (d, *J* = 8.2 Hz, 1H, Ar), 8.13–8.16 (m, 2H, Ar,), 8.18–8.22 (m, 2H, Ar,), 9.32 (br s, 1H, NH).^13^C NMR (101 MHz, CDCl_3_): δ (ppm) 21.26, 36.04, 113.98, 116.49, 119.91, 124.72, 124.84, 125.43, 126.68, 127.54, 127.62, 128.13, 128.69, 128.90, 129.35, 130.55, 130.93, 131.76, 134.09, 135.60, 136.10, 137.93, 138.61, 165.24, 167.45, 167.79, 171.41. Anal. calcd. for C_29_H_23_N_3_OS: C 75.46, H 5.02, N 9.10, S 6.95; found C 75.23, H 5.29, N 8.81, S 6.68.
*2-{[4-(4-Chlorophenyl)-6-(naphthalen-1-yl)pyrimidin-2-yl]thio}-N-(m-tolyl)acetamide (***2g***)*. Yield: 20%. Mp: 103–107 °C. ^1^H NMR (200 MHz, CDCl_3_): δ (ppm) 2.06 (s, 3H, CH_3_), 4.00 (s, 2H, CH_2_), 6.60 (s, 1H, Ar), 6.75–6.76 (m, 1H, Ar), 6.96–6.99 (m, 2H, Ar), 7.51–7.65 and 7.75–7.78 (2m, 7H, 6Ar + H-5), 7.98 (d, *J* = 8.0 Hz, 1H, Ar), 8.04–8.06 and 8.10–8.12 (2 m, 3H, Ar), 8.19 (d, *J* = 7.6 Hz, Ar), 9.25 (br s, 1H, NH). ^13^C NMR (101 MHz, CDCl_3_): δ (ppm) 21.26, 36.05, 113.63, 116.49, 119.88, 124.73, 124.82, 125.43, 126.74, 127.61, 128.16, 128.73, 128.89, 128.95, 129.57, 130.49, 131.05, 134.09, 134.44, 135.46, 137.82, 138.13, 138.67, 164.04, 167.25, 167.91, 171.45. Anal. calcd. for C_29_H_22_ClN_3_OS: C 70.22, H 4.47, N 8.47, S 6.46; found 70.45, H 4.73, N 8.15, S 6.80.
*2-{[4-(4-Fluorophenyl)-6-(1-naphthyl)pyrimidin-2-yl]thio}-N-(3-methylphenyl)acetamide (***2h***)*. Yield: 55%. Mp: 145–147 °C. ^1^H NMR (400 MHz, CDCl_3_): δ (ppm) 2.04 (s, 3H, CH_3_), 3.97 (s, 2H, CH_2_), 6.58 (br s, 1H, Ar), 6.74 (d, *J* = 6.0 Hz, 1H, Ar), 6.92–7.00 (m, 2H, Ar), 7.18–7.25 (m, 2H, Ar), 7.51–7.63 (m, 3H, Ar), 7.70–7.77 (m, 2H, 1H Ar + H-5), 7.94–8.00 (m, 1H, Ar), 8.03 (d, *J* = 8.2 Hz, 1H Ar), 8.13–8.21 (m, 3H Ar), 9.20 (br s, 1H, NH). ^13^C NMR (101 MHz, CDCl_3_): δ (ppm) 21.26, 36.04, 113.50, 116.30, 116.49, 116.51, 119.89, 124.76, 124.79, 125.41, 126.70, 127.56, 128.11, 128.70, 128.93, 129.73, 129.82, 130.51, 130.98, 132.14, 132.18, 134.08, 135.52, 137.85, 138.64, 164.08, 165.13 (d, *J*_C-F_ = 252 Hz), 167.27, 167.76, 171.34. Anal. calcd. for C_29_H_22_FN_3_OS: C 72.63, H 4.62, N 8.76, S 6.69; found C 72.49, H 4.36, N 8.42, S 6.93.
*2-{[4-Phenyl-6-(phenylthio)pyrimidin-2-yl]thio}-N-(m-tolyl)acetamide (***2i***)*. Yield: 27%. Mp: 127–128 °C. ^1^H NMR (400 MHz, CDCl_3_): δ (ppm) 2.33 (s, 3H, CH_3_), 3.68 (s, 2H, CH_2_), 6.89 (d, *J* = 7.4 Hz, 1H, Ar), 7.18 (t, *J* = 7.8 Hz, 1H, Ar), 7.25–7.26 (m, 1H, Ar), 7.30–7.31 (m, 2H, 1H Ar + H-5), 7.35–7.49 (m, 6H, Ar), 7.63–7.70 (m, 2H Ar), 7.90 (dd, *J* = 8.3, 1.3 Hz, 2H Ar), 8.57 (br s, 1H, NH). ^13^C NMR (100 MHz, CDCl_3_): δ (ppm) 21.58, 33.57, 110.17, 117.15, 120.71, 125.12, 127.39, 128.77, 129.01, 129.17, 129.37, 129.76, 131.56, 135.42, 135.62, 137.79, 138.82, 162.84, 166.99, 169.30, 172.16. Anal. calcd. for C_25_H_21_N_3_OS_2_: C 67.69, H 4.77, N 9.47, S 14.46; found C 67.75, H 4.77, N 9.47, S 14.10.
*2-{[4-(4-Chlorophenyl)-6-(phenylthio)pyrimidin-2-yl]thio}-N-(m-tolyl)acetamide (***2j***)*. Yield: 20%. Mp: 150–154 °C. ^1^H NMR (400 MHz, CDCl_3_): δ (ppm) 2.34 (s, 3H, CH_3_), 3.70 (s, 2H, CH_2_), 6.91 (d, *J* = 7.2 Hz, 1H, Ar), 7.17–7.21 and 7.25–7.31 (2 m, 4H, 3Ar + H-5), 7.37–7.46 (m, 5H, Ar), 7.67 (d, *J* = 7.2 Hz, 2H, Ar), 7.85 (d, *J* = 8.4 Hz, 2H, Ar), 8.61 (br s, 1H, NH).^13^C NMR (101 MHz, CDCl_3_): δ (ppm) 21.59, 33.58, 109.91, 117.15, 120.71, 125.17, 128.65, 128.80, 129.30, 129.42, 129.86, 133.87, 135.66, 137.75, 137.89, 138.86, 161.57, 166.88, 169.61, 172.35. Anal. calcd. for C_25_H_20_ClN_3_OS_2_: C 62.82, H 4.22, N 8.79, S 13.41; found C 62.51, H 4.38, N 9.00, S 13.46.
*2-{[4-(4-Fluorophenyl)-6-(phenylthio)pyrimidin-2-yl]thio}-N-(m-tolyl))acetamide (***2k***)*. Yield: 20%. Mp: 146–147 °C. ^1^H NMR (200 MHz, CDCl_3_): δ (ppm) 2.34 (s, 3H, CH_3_), 3.70 (s, 2H, CH_2_), 6.91 (d, *J* = 7.2 Hz, 1H, Ar), 7.10–7.13 and 7.17–7.21 (2m, 3H, Ar), 7.25–7.27 (m, 2H, Ar), 7.31 (s, 1H, H-5), 7.37–7.46 (m, 3H, Ar), 7.67 (d, *J* = 7.2 Hz, 2H, Ar), 7.90–7.93 (m, 2H, Ar), 8.53 (br s, 1H, NH).^13^C-NMR (101 MHz, CDCl_3_): δ (ppm) 21.59, 33.57, 116.03, 116.25,. 117.15, 120.71, 125.16, 128.80, 129.07, 129.42, 129.50, 129.59, 129.84, 130.99, 131.59, 135.66, 137.77, 138.86, 161.68, 165.00 (d, *J*_C-F_ = 254 Hz), 166.94, 169.45, 172.25. Anal. calcd. for C_25_H_20_FN_3_OS_2_: C 65.05, H 4.37, N 9.10, S 13.89; found C 65.03, H 4.31, N 9.28, S 13.89.
*2-{[2-oxo-2-(m-tolylamino)ethyl]thio}-N,6-diphenylpyrimidine-4-carboxamide (***2l***)*. Yield: 62%. Mp: 181–182 °C. H^1^ NMR (400 MHz, DMSO-*d*_6_): δ (ppm) 2.19 (s, 3H, CH_3_), 4.21 (s, 2H, CH_2_), 6.83 (d, *J* = 7.6 Hz, 1H, Ar), 7.13–7.19 (m, 3H, Ar), 7.36–7.43 and 7.46–7.50 (2m, 5H, Ar), 7.57 (t, *J* = 7.2 Hz, 1H, Ar), 7.88 (d, *J* = 8.0 Hz, 2H, Ar), 8.24–8.26 (m, 3H, 2H Ar + H-5), 10.29 and 10.52 (2 br s, 1H, 2 NH). ^13^C NMR (101 MHz, DMSO-*d*_6_): δ (ppm) 21.63, 36.58, 110.30, 116.98, 120.34, 121.08, 124.70, 125.15, 128.13, 129.14, 129.36, 129.65, 132.54, 135.61, 138.31, 138.48, 139.36, 158.89, 161.51, 166.33, 167.41, 171.26. Anal. calcd. for C_26_H_22_N_4_O_2_S: C 68.70, H 4.88, N 12.33, S 7.05; found C 68.40, H 5.18, N 12.14, S 6.70.

#### 2.1.16. General Procedure for the Synthesis of Compounds **2m**,**n**

A round-bottom flask was charged using the appropriate intermediate **8b**,**c** (1.00 mmol), anhydrous K_2_CO_3_ (0.28 g, 2.00 mmol), CuI (19.05 mg, 0.10 mmol), and L-proline (34.54 mg, 0.30 mmol). The flask was then evacuated and filled with nitrogen. A measure of 2-mercapto-*N*-(*m*-tolyl)acetamide **14** (0.20 g, 1.10 mmol) dissolved in anhydrous DME (5 mL) was introduced via syringe, and the mixture was stirred at reflux under nitrogen atmosphere for 24 h. Then, the reaction was extracted with CH_2_Cl_2_ (3 × 30 mL), and the organic phase was washed with saturated NaCl solution, dried over Na_2_SO_4_, filtered, and concentrated under reduced pressure. The crude product was purified by column chromatography using CH_2_Cl_2_ as eluent to obtain the final compounds **2m**,**n** as off-white solids.

*2-{[4-Phenyl-6-(phenylthio)pyridin-2-yl]thio}-N-(m-tolyl)acetamide (***2m***)*. Yield: 20%. Mp: 122–123 °C. ^1^H NMR (200 MHz, CDCl_3_): δ (ppm) 2.33 (s, 3H, CH_3_), 3.76 (s, 2H, CH_2_), 6.89 (d, *J* = 7.2 Hz, 1H, Ar), 7.00 (s, 1H, Ar), 7.17–7.21 (m, 2H, Ar), 7.39–7.45 and 7.62–7.63 (2m, 12H, 10H Ar + 2H pyr), 9.34 (br s, 1H, NH). ^13^C NMR (101 MHz, CDCl_3_): δ (ppm) 21.41, 34.58, 116.31. 116.68, 116.88, 120.45, 124.65, 126.94, 128.58, 129.10, 129.60, 129.74, 129.87, 135.00, 136.98, 138.16, 138.59, 146.18, 150.29, 161.14, 167.50, 168.05. Anal. calcd. for C_26_H_22_N_2_OS_2_: C 70.56, H 5.01, N 6.33, S 14.49; found C 70.52, H 5.31, N 6.07, S 14.84.
*2-{[4-(4-chlorophenyl)-6-(phenylthio)pyridin-2-yl]thio}-N-(m-tolyl)acetamide (***2n***)*. Yield: 20%. Mp: 93–95 °C^1^H NMR (200 MHz, CDCl_3_): δ (ppm) 2.32 (s, 3H, CH_3_), 3.71 (s, 2H, CH_2_), 6.87–6.91 (m, 2H, Ar), 7.12 (s, 1H, Ar), 7.17 (t, *J* = 7.16 Hz, 1H, Ar), 7.35–7.51 (m, 9H, 7H Ar + 2H pyr), 7.61 (dd, *J* = 7.6, 1.6 Hz, 2H, Ar) 9.19 (br s, 1H, NH). ^13^C NMR (101 MHz, CDCl_3_): δ (ppm) 167.97, 161.68, 158.67, 148.98, 138.78, 138.27, 135.99, 135.63, 135.18, 129.90, 129.80, 129.49, 128.75, 128.43, 128.33, 124.81, 120.54, 118.49, 116.95, 116.34, 34.52, 21.60. Anal. calcd. for C_26_H_21_ClN_2_OS_2_ C 65.46, H 4.44, N 5.87, S 13.44, found C 65.13, H 4.64, N 6.20, S 13.58.

#### 2.1.17. General Procedure for the Synthesis of Compounds **15a**,**b**

To a solution of the opportune intermediates **5b**,**d** (0.50 mmol) in ACN (5 mL), ethyl thioglycolate (0.08 mL, 0.75 mmol) and Cs_2_CO_3_ (0.50 g, 1.5 mmol) were added. The reaction was stirred at 30–40 °C for 1 h. Then, the reaction mixture was filtered, and the solvent removed under reduced pressure. The crude residue was purified by flash chromatography using PE (bp 40–60 °C)/AcOEt (97:3) as the eluent to afford the compounds **15a**,**b** as white solids.

*Ethyl 2-{[4,6-bis(4-fluorophenyl)pyrimidin-2-yl]thio}acetate (***15a***)*. Yield: 68%.^1^H NMR (400 MHz, CDCl_3_): δ (ppm) 1.25 (t, *J* = 7.1 Hz, 3H, CH_3_), 3.99 (d, *J* = 1.3 Hz, 2H, S-CH_2_), 4.19 (q, *J* = 7.1 Hz, 2H, O-CH_2_), 7.17–7.23 (m, 4H, *p*F-Ar), 7.67 (d, *J* = 1.3 Hz, 1H, CH-Pyr), 8.18–8.10 (m, 4H, *p*F-Ar). Anal. calcd. for C_20_H_16_F_2_N_2_O_2_S: C, 62.17; H, 4.17; N, 7.25; S, 8.30; found C, 62.20; H, 4.17; N, 7.24; S, 8.23.
*Ethyl 2-{[4-(4-fluorophenyl)-6-phenylpyrimidin-2-yl]thio}acetate (***15b***)*. Yield: 76%. ^1^H NMR (400 MHz, CDCl_3_): δ (ppm) 1.24 (t, *J* = 7.1 Hz, 3H, CH_3_), 4.01 (s, 2H, S-CH_2_), 4.19 (q, *J* = 7.1 Hz, 2H, O-CH_2_), 7.08–7.25 (m, 2H, Ar), 7.48–7.52 (m, 3H, Ar), 7.73 (s, 1H, CH-Pyr), 8.10–8.16 (m, 4H, *p*F-Ar). Anal. calcd. for C_20_H_17_FN_2_O_2_S: C, 65.20; H, 4.65; N, 7.60; S, 8.70; found C, 65.23; H, 4.62; N, 7.63; S, 8.85.

#### 2.1.18. General Procedure for the Synthesis of Compounds **15c**,**d**

To a solution of the opportune intermediates **8a**,**d** (0.5 mmol) in DMF (10 mL), ethyl thioglycolate (0.16 mL, 1.5 mmol) and Cs_2_CO_3_ (0.81 g, 2.5 mmol) were added. The reaction was stirred at 100 °C (±5 °C) for 6 h. Then, the reaction mixture was filtered, and the residual solvent concentrated under reduced pressure. Then, the product was extracted with DCM. The organic phase was washed with H_2_O and dried over Na_2_SO_4_. The crude residue was purified by column chromatography using PE (bp 40–60 °C)/AcOEt (97:3) as the eluent to obtain the compounds **15c**,**d** as white solids.

*Ethyl 2-{[4,6-diphenylpyridin-2-yl]thio}acetate (***15c***)*. Yield: 25%. ^1^H NMR (400 MHz, CDCl_3_): δ (ppm) 1.25 (t, *J* = 7.1 Hz, 3H, CH_3_), 4.08 (s, 2H, S-CH_2_), 4.20 (q, *J* = 7.1 Hz, 2H, O-CH_2_), 7.37 (d, *J* = 1.4 Hz, 1H, CH-Py), 7.40–7.53 (m, 6H, Ar), 7.58–7.80 (m, 3H, Ar + CH-Py), 8.00–8.18 (m, 2H, Ar). Anal. calcd. for C_21_H_19_NO_2_S: C, 72.18; H, 5.48; N, 4.01; S, 9.17; found C, 72.20; H, 5.50; N, 3.98; S, 9.22.
*Ethyl 2-{[6-(4-fluorophenyl)-4-phenylpyridin-2-yl]thio}acetate (***15d***)*. Yield: 40%.^1^H NMR (400 MHz, DMSO-*d*_6_): δ (ppm) 1.12 (t, *J* = 7.1 Hz, 3H, CH3), 4.06 (q, *J* = 7.1 Hz, 2H, O-CH_2_), 4.10 (s, 2H, S-CH_2_), 7.30 (t, *J* = 8.8 Hz, 2H, Ar), 7.40–7.55 (m, 3H, Ar), 7.59 (d, *J* = 1.3 Hz, 1H, CH-Py), 7.88 (dd, *J* = 8.0, 1.6 Hz, 2H, *p*F-Ar), 7.94 (d, *J* = 1.3 Hz, 1H, CH-Py), 8.27–8.19 (m, 2H, *p*F-Ar). Anal. calcd. for C_21_H_18_FNO_2_S: C, 68.65; H, 4.94; N, 3.81; S, 8.73; found C, 68.67; H, 4.96; N, 3.84; S, 8.75.

#### 2.1.19. General Procedure for the Synthesis of Compounds **16a**–**d**

To a solution of the opportune intermediates **16a**–**d** (0.50 mmol) in THF (5 mL), a solution of 5% *w*/*v* NaOH was added. The reaction was stirred at room temperature for 16 h. Then, the reaction mixture was quenched with 1 N HCl and the product was extracted with DCM (3 × 10 mL). The organic layer was washed with H_2_O and dried over Na_2_SO_4_. The solvent was removed under reduced pressure, affording pale yellow solids with a quantitative yield that were used in the next step without any purification.

#### 2.1.20. General Procedure for the Synthesis of Compounds **3a**–**n**

To a stirred solution of opportune intermediates **16a**–**d** (50 mg, 0.14 mmol) in dry DMF (0.5 mL) at 0° C under nitrogen atmosphere, the appropriate L-amino acid methyl ester (0.15 mmol), EDC (0.03 g, 0.14 mmol), HOBt (0.02 mg, 0.14 mmol), and dry DIPEA (0.03 mL, 0.20 mmol) were added. The mixture was stirred for 30 min at 0 °C and then for 4–6 h at room temperature. The reaction was quenched with H_2_O and extracted with AcOEt (3 × 10 mL). The organic phase was washed with H_2_O, dried over Na_2_SO_4_, filtered, and concentrated under reduced pressure. The crude residue was purified by column chromatography using PE (bp 40–60 °C)/AcOEt (1:1) as the eluent to give the desired final compounds **3a**–**n**.

*Methyl (2-((4-(4-fluorophenyl)-6-phenylpyrimidin-2-yl)thio)acetyl)-L-valinate (***3a***)**.*** Yield: 48%. Mp: 152–153 °C. ^1^H NMR (400 MHz, CDCl_3_): δ (ppm) 0.58 (d, *J* = 6.8 Hz, 3H, CH_3_-Val), 0.68 (d, *J* = 6.8 Hz, 3H, CH_3_-Val), 1.94–1.99 (m, 1H, CH-Val), 3.53 (s, 3H, OCH_3_), 3.92–4.03 (m, 2H, S-CH_2_), 4.47–4.51 (m, 1H, CH-Val), 7.18–7.23 (m, 2H, Ar), 7.37 (d, *J* = 9.0 Hz, 1H, C=O-NH), 7.51–7.54 (m, 3H, Ar), 7.80 (s, 1H, CH-Pyr), 8.11–8.18 (m, 4H, Ar). ^13^C NMR (101 MHz, DMSO-*d*_6_) δ18.67, 19.35, 30.66, 34.84, 52.20, 58.28, 108.61, 116.29, 116.50, 128.04, 129.44, 130.65, 131.99, 132.90, 136.32, 163.50, 163.71, 164.74 (d, *J*_C-F_ = 248 Hz), 164.85, 165.98, 168.46, 171.19, 172.39. Anal. calcd. for C_24_H_24_FN_3_O_3_S: C, 63.56; H, 5.33; N, 9.27; S, 7.07; found C, 63.56; H, 5.33; N, 9.28; S, 7.07.
*Methyl (2-((4-(4-fluorophenyl)-6-phenylpyrimidin-2-yl)thio)acetyl)-L-isoleucinate (***3b***)*. Yield: 60%. Mp: 94–95 °C.^1^H NMR (400 MHz, CDCl_3_): δ (ppm) 0.57–0.65 (m, 6H, (CH_3_)_2_-Iso), 0.73–0.88(m, 2H, CH_2_-Iso), 1.08–1.18 (m,1H, CH-Iso), 3.53 (s, 3H, OCH_3_), 3.90 (d, *J* = 15.7 Hz, 1H, S-CH), 4.01 (d, *J* = 15.7 Hz, 1H, S-CH), 4.49–4.53 (m, 1H), 7.19–7.23 (m, 2H, Ar), 7.38 (d, *J* = 8.9 Hz, 1H, C=O-NH), 7.51–7.55 (m, 3H, Ar), 7.80 (s, 1H, CH-Pyr), 8.11–8.18 (m, 4H, *p*F-Ar). ^13^C NMR (101 MHz, DMSO-*d*_6_) δ 11.57, 15.77, 25.23, 34.83, 37.09, 52.16, 57.23, 108.62, 116.40, 128.04, 129.44, 130.61, 131.99, 132.87, 136.31, 163.72, 164.75 (d, *J* _C-F_ = 249.0 Hz), 164.85, 168.37, 171.17, 172.37. Anal. calcd. for C_25_H_26_FN_3_O_3_S: C, 64.22; H, 5.61; N, 8.99; S, 6.86; found C, 64.27; H, 5.58; N, 9.01; S, 6.87.
*Methyl (2-((4-(4-fluorophenyl)-6-phenylpyrimidin-2-yl)thio)acetyl)-L-tyrosinate (***3c***)*. Yield: 30%. Mp: 167–168 °C. ^1^H NMR (400 MHz, CDCl_3_): δ (ppm) 2.79 (dd, *J* = 14.1, 6.8 Hz, 1H, CH-Tyr), 2.90 (dd, *J* = 14.1, 5.4 Hz, 1H, CH-Tyr), 3.56 (s, 3H, OCH_3_), 3.84–3.94 (m, 2H, S-CH_2_), 4.76–4.81 (m, 1H, CH), 6.33 (d, *J* = 8.5 Hz, 2H, Ar-Tyr), 6.65 (m, 2H, d, *J* = 8.5 Hz, 2H, Ar-Tyr), 7.17–7.21 (m, 2H, Ar), 7.32 (d, *J* = 8.0 Hz, 1H, C=O-NH), 7.52–7.53 (m, 3H, Ar), 7.77 (s, 1H, CH-Pyr), 8.14–8.09 (m, 4H, Ar). ^13^C NMR (101 MHz, CDCl_3_) δ 34.68, 36.86, 52.33, 53.48, 108.42, 115.30, 116.13, 116.35, 127.41, 129.20, 129.62, 130.01, 131.54, 132.39, 136.18, 154.55, 164.06, 164.95 (d, *J*_C-F_ = 251.9 Hz), 165.24, 169.00, 170.37, 171.72. Anal. calcd. for C_28_H_24_FN_3_O_4_S: C, 64.98; H, 4.67; N, 8.12; S, 6.19; found C 65.00; H, 4.68; N, 8.12; S, 6.20.
*Methyl (2-((4-(4-fluorophenyl)-6-phenylpyrimidin-2-yl)thio)acetyl)-L-histidinate (***3d***)*. Yield: 55%. Mp: 77–78 °C. ^1^H NMR (400 MHz, DMSO-*d*_6_): δ (ppm) 2.81–2.86 (m, 2H, CH_2_-His), 3.44 (s, 3H, OCH_3_), 4.01 (s, 2H, S-CH_2_), 4.42–4.55(m, 1H, CH), 6.73 (d, *J* = 1.2 Hz, 1H, CH-ArHis), 7.29–7.39 (m, 2H, Ar), 7.47 (d, *J* = 1.2 Hz, 1H, CH-ArHis), 7.51–7.56 (m, 3H, Ar), 7.63 (d, *J* = 8.4 Hz, 1H, C=O-NH), 7.90 (d, *J* = 7.5 Hz, 1H, NH-His tautomer), 8.24–8.33 (m, 3H, Ar and CH-Pyr), 8.35–8.43 (m, 2H, *p*F-Ar), 8.64 (d, *J* = 7.5 Hz, 1H, NH-His). ^13^C NMR (101 MHz, DMSO-*d*_6_) δ 29.50, 34.89, 52.29, 53.25, 108.63, 110.36, 116.29, 119.50, 124.69, 127.16, 128.03, 129.44, 130.58, 131.98, 135.37, 136.33, 163.69, 164.74 (d, *J*_C-F_ = 249.0 Hz), 164.83, 168.16, 170.94, 172.23. Anal. calcd. for C_25_H_22_FN_5_O_3_S: C, 61.09; H, 4.51; N, 14.25; S, 6.52; found C, 61.10; H, 4.50; N, 14.27; S, 6.52.
*Methyl (2-((4,6-bis(4-fluorophenyl)pyrimidin-2-yl)thio)acetyl)-L-valinate (***3e***)*. Yield: 60%. Mp: 174–175 °C. ^1^H NMR (400 MHz, CDCl_3_): δ (ppm) 0.77 (t, *J* = 6.4 Hz, 6H, (CH_3_)_2_-Val), 1.92–2.01 (m, 1H, CH-Val), 3.53 (s, 3H, OCH_3_), 4.01–4.10 (m, 2H, S-CH_2_), 4.14–4.19 (m, 1H, CH), 7.33–7.38 (m, 4H, *p*F-Ar), 8.29 (s, 1H, CH-Pyr), 8.36–8.48 (m, 4H, Ar). ^13^C NMR (101 MHz, DMSO-*d*_6_) δ 18.65, 19.34, 30.66, 34.84, 52.19, 58.26, 108.44, 116.40, 130.61, 132.82, 163.75, 164.77 (d, *J*_C-F_ = 249.5 Hz), 168.45, 171.19, 172.39. Anal. calcd. for C_24_H_23_F_2_N_3_O_3_S: C, 61.13; H, 4.92; N, 8.91; S, 6.80; found C, 61.00; H, 4.90; N, 8.89; S, 6.81.
*Methyl (2-((4,6-bis(4-fluorophenyl)pyrimidin-2-yl)thio)acetyl)-L-isoleucinate (***3f***)*. Yield: 64%. Mp: 150–151 °C. ^1^H NMR (400 MHz, CDCl_3_): δ (ppm) 0.61–0.64 (m, 6H, (CH_3_)_2_-Iso), 0.76–0.87 (m, 2H, CH_2_-Iso), 1.09–1.19 (m, 1H, CH-Iso), 3.54 (s, 3H, OCH_3_), 3.89 (d, *J* = 15.8 Hz, 1H, S-CH), 4.00 (d, *J* = 15.8 Hz, 1H, S-CH), 4.50–4.53 (m, 1H, CH), 7.18–7.24 (m, 4H, Ar), 7.33 (d, *J* = 9.1 Hz, 1H, C=O-NH), 7.75 (s, 1H, CH-Pyr), 8.13–8.18 (m, 4H, Ar). ^13^C NMR (101 MHz, DMSO-*d*_6_) δ 11.54, 15.76, 25.20, 34.83, 37.09, 52.16, 57.22, 116.29, 116.50, 130.58, 130.66, 132.82, 163.76, 164.77 (d, *J*_C-F_ = 249.5 Hz), 168.37, 171.17, 172.37. Anal. calcd. for C_25_H_25_F_2_N_3_O_3_S: C, 61.84; H, 5.19; N, 8.65; S, 6.60; found C, 61.53; H, 5.25; N, 8.80; S, 6.40.
*Methyl (2-((4,6-bis(4-fluorophenyl)pyrimidin-2-yl)thio)acetyl)-L-tyrosinate (***3g***)*. Yield: 35%. ^1^H NMR (400 MHz, DMSO-*d*_6_): δ (ppm) 2.72–2.85 (m, 2H, CH_2_-Tyr), 3.45 (s, 3H, OCH_3_), 3.99 (d, *J* = 2.9 Hz, 2H, S-CH_2_), 4.35–4.41 (m, 1H, CH), 6.5–6.52 (m, 2H, Ar-Tyr), 6.84–6.86 (m, 2H, Ar-Tyr), 7.34–7.38 (m, 4H, Ar), 8.30 (s, 1H, CH-Pyr), 8.38–8.41 (m,4H, Ar), 8.55 (d, *J* = 7.6 Hz, 1H, C=O-NH), 9.16 (d, *J* = 1.0 Hz, 1H, OH). ^13^C NMR (101 MHz, DMSO-*d*_6_) δ 34.77, 36.85, 52.37, 53.46, 108.06, 115.30, 116.18, 116.39, 127.47, 129.63, 130.03, 132.21, 154.53, 164.10, 165.00 (d, *J*_C-F_ = 252.9 Hz), 168.84, 170.33, 171.73. Anal. calcd. for C_28_H_23_F_2_N_3_O_4_S C, 62.79; H, 4.33; N, 7.85; S, 5.99; found C, 62.83; H, 4.37; N, 7.86; S, 6.01.
*Methyl (2-((4,6-bis(4-fluorophenyl)pyrimidin-2-yl)thio)acetyl)-L-histidinate (***3h***)*. Yield: 43%. Mp: 107–108 °C. ^1^H NMR (400 MHz, DMSO-*d*_6_): δ (ppm) 2.76–2.92(m, 2H, CH_2_-His), 3.45 (s, 3H, OCH_3_), 4.00 (s, 2H, S-CH_2_), 4.44–4.54 (m, 1H, CH), 6.72 (d, *J* = 1.4 Hz, 1H, CH-ArHis), 7.36 (t, *J* = 8.9 Hz, 4H, Ar), 7.46 (d, *J* = 1.4 Hz, 1H, CH-ArHis), 7.61 (d, *J* = 8.3 Hz, 1H, C=O-NH), 7.89 (d, *J* = 8.4, 1H, NH-His tautomer), 8.30 (s, 1H, CH-Pyr), 8.35–8.47 (m, 4H, Ar). ^13^C NMR (101 MHz, DMSO-*d*_6_) δ 29.43, 34.89, 52.30, 53.21, 110.36, 116.40, 119.49, 124.68, 127.13, 130.58, 132.84, 135.36, 163.72, 164.75 (d, *J* = 249.0 Hz), 168.18, 170.93, 172.22. nal. calcd. for C_25_H_21_F_2_N_5_O_3_S: C, 58.93; H, 4.15; N, 13.74; S, 6.29; found C, 59.01; H, 4.17; N, 14.01; S, 6.34.
*Methyl (2-((4,6-diphenylpyridin-2-yl)thio)acetyl)-L-valinate (***3i***)*. Yield: 22%. Mp: 63–64 °C. ^1^H NMR (400 MHz, DMSO-*d*_6_): δ (ppm) 0.75–0.79 (m, 6H, (CH_3_)_2_), 1.90–1.99 (m, 1H, CH-Val), 3.53 (s, 3H, OCH_3_), 4.01–4.10 (m, 2H, S-CH_2_), 4.15–4.18 (m, 1H, CH), 7.42–7.52 (m, 6H, Ar), 7.59 (d, *J* = 1.4 Hz, 1H, CH-Py), 7.87–7.89 (m, 2H, Ar), 7.92 (d, *J* = 1.3 Hz, 1H, CH-Py), 8.18–8.20 (m, 2H, Ar), 8.40 (d, *J* = 8.2 Hz, 1H, C=O-NH). ^13^C NMR (101 MHz, DMSO-*d*_6_): δ (ppm) 17.94, 30.88, 34.53, 53.23, 56.29, 116.84, 122.47, 128.00, 128.16, 128.26, 129.42, 129.69, 129.91, 137.65, 138.57, 147.39, 156.02, 157.06, 168.68, 173.33. Anal. calcd. for C_25_H_26_N_2_O_3_S C, 69.10; H, 6.03; N, 6.45; S, 7.38; found C, 69.13; H, 6.02; N, 6.45; S, 7.40.
*Methyl (2-((4,6-diphenylpyridin-2-yl)thio)acetyl)-L-isoleucinate (***3j***)*. Yield: 25%. Mp: 70–71 °C. ^1^H NMR (400 MHz, DMSO-*d*_6_): δ (ppm) 0.65–0.71 (m, 6H, 2xCH_3_, 0.97–1.06 (m, 1H, CH-Iso), 1.19–1.29 (m, 1H, CH-Iso), 1.63–1.70 (m, 1H, CH-Iso), 3.52 (s, 3H, OCH_3_), 3.99–4.12 (m, 2H, S-CH_2_), 4.18–4.21 (m, Hz, 1H CH-Iso), 7.43–7.52 (m, 6H, Ar), 7.58 (d, *J* = 1.4 Hz, 1H, CH-Py), 7.87–7.89 (m, 2H, Ar), 7.93 (d, *J* = 1.4 Hz, 1H, CH-Py), 8.18–8.20 (m, 2H, Ar), 8.39 (d, *J* = 8.1 Hz, 1H, C=O-NH). ^13^C NMR (101 MHz, DMSO-*d*_6_) δ 11.60, 15.78, 25.19, 33.67, 37.07, 52.17, 57.18, 114.70, 117.79, 127.47, 127.74, 129.22, 129.64, 129.90, 130.02, 137.55, 138.66, 149.46, 157.02, 158.93, 168.72, 172.30. Anal. calcd. for C_26_H_28_N_2_O_3_S: C, 69.62; H, 6.29; N, 6.25; S, 7.15; found C, 69.64; H, 6.29; N, 6.23; S, 7.15.
*Methyl (2-((4,6-diphenylpyridin-2-yl)thio)acetyl)-L-tyrosinate (***3k***)*. Yield: 27%. Mp: 111–112 °C. ^1^H NMR (400 MHz, DMSO-*d*_6_): δ (ppm) 2.69–2.85 (m, 2H, CH_2-_Tyr), 3.46 (s, 3H, OCH_3_), 4.00 (s, 2H, S-CH_2_), 4.35–4.40 (m, 1H, CH-Tyr), 6.53 (d, *J* = 8.4 Hz, 2H, Ar-Tyr), 6.84(d, *J* = 8.4 Hz, 2H, Ar-Tyr), 7.41–7.53 (m, 6H, Ar), 7.57 (d, *J* = 1.4 Hz, 1H, CH-Py), 7.87–7.90 (m, 2H, Ar), 7.94 (d, *J* = 1.4 Hz, 1H, CH-Py), 8.18–8.20 (m, 2H, Ar), 8.53 (d, *J* = 7.6 Hz, 1H, C=O-NH), 9.17 (s, 1H, OH). ^13^C NMR (101 MHz, DMSO-*d*_6_) δ 33.64, 36.71, 52.28, 54.79, 114.72, 115.56, 117.85, 127.26, 127.45, 127.76, 129.23, 129.66, 129.92, 130.01, 130.45, 137.58, 138.65, 149.45, 156.53, 156.95, 158.71, 168.46, 172.33. Anal. calcd. for C_29_H_26_N_2_O_4_S: C, 69.86; H, 5.26; N, 5.62; S, 6.43; found C, 69.87; H, 5.26; N, 5.61; S, 6.42.
*Methyl (2-((6-(4-fluorophenyl)-4-phenylpyridin-2-yl)thio)acetyl)-L-isoleucinate (***3l***)*. Yield: 27%. Mp: 76–77 °C. ^1^H NMR (400 MHz, DMSO-*d*_6_): δ (ppm) 0.55–0.75 (m, 6H (CH_3_)_2_), 0.95–1.07 (m, 1H, CH-Iso), 1.19–1.29 (m, 1H, CH-Iso), 1.63–1.70 (m, 1H, CH), 3.53 (s, 3H, OCH_3_), 4.00 (d, *J* = 15.1 Hz, 1H, S-CH), 4.10 (d, *J* = 15.1 Hz, 1H, S-CH), 4.19–4.21 (m, 1H, CH), 7.25–7.31 (m, 2H, Ar), 7.44–7.52 (m, 3H, Ar), 7.58 (d, *J* = 1.3 Hz, 1H, CH-Py), 7.80–7.92 (m, 2H, Ar), 7.94 (d, *J* = 1.3 Hz, 1H, CH-Py), 8.25–8.29 (m, 2H, *p*F-Ar), 8.40 (d, *J* = 8.2 Hz, 1H, C=O-NH). ^13^C NMR (101 MHz, DMSO-*d*_6_) δ 11.58, 15.76, 25.15, 33.67, 37.07, 52.18, 57.16, 114.53, 116.02, 117.69, 127.77, 129.63, 130.05, 135.18, 137.47, 149.49, 155.97, 158.96, 163.58 (d, *J* _C-F_ = 246.6 Hz), 168.70, 172.34. Anal. calcd. for C_26_H_27_FN_2_O_3_S: C, 66.93; H, 5.83; N, 6.00; S, 6.87; found C, 67.00; H, 5.82; N, 6.05; S, 6.89.
*Methyl (2-((6-(4-fluorophenyl)-4-phenylpyridin-2-yl)thio)acetyl)-L-tyrosinate (***3m***)*. Yield: 30%. Mp: 151–152 °C. ^1^H NMR (400 MHz, DMSO-*d*_6_): δ (ppm) 2.71–2.83 (m, 2H, CH_2_-Tyr), 3.47 (s, 3H, OCH_3_), 3.99 (d, *J* = 1.5 Hz, 2H, S-CH_2_), 4.35–4.40(m, 1H, CH), 6.53 (d, *J* = 8.4 Hz, 2H, Ar-Tyr), 6.84 (d, *J* = 8.0 Hz, 2H, Ar-Tyr), 7.28 (t, *J* = 8.8 Hz, 2H, Ar), 7.44–7.53 (m, 3H, Ar), 7.57 (d, *J* = 1.3 Hz, 1H, CH-Py), 7.87–7.90 (m, 2H, Ar), 7.94 (d, *J* = 1.4 Hz, 1H, CH-Py), 8.24–8.27(m, 2H, *p*F-Ar), 8.51 (d, *J* = 7.6 Hz, 1H, C=O-NH), 9.17 (s, 1H, OH). ^13^C NMR (101 MHz, DMSO-*d*_6_) δ 33.64, 36.69, 52.28, 54.76, 114.56, 115.55, 115.92, 116.13, 117.75, 127.27, 127.78, 129.77, 130.04, 130.44, 135.14, 137.51, 149.50, 155.91, 156.53, 158.73, 163.58 (d, *J*_C-F_ = 246.6 Hz), 168.46, 172.34. Anal. calcd. for C_29_H_25_FN_2_O_4_S: C, 67.43; H, 4.88; N, 5.42; S, 6.21; found C, 67.48; H, 4.92; N, 5.42; S, 6.23.
*Methyl (2-((6-(4-fluorophenyl)-4-phenylpyridin-2-yl)thio)acetyl)-L-histidinate (***3n***)*. Yield: 20%. Mp: 155–156 °C. ^1^H NMR (400 MHz, DMSO-*d*_6_): δ (ppm) 2.74–2.90 (m, 2H, CH_2_-His), 3.45 (s, 3H, OCH_3_), 4.00 (s, 2H, S-CH_2_), 4.28–4.60 (m, 1H, CH), 6.70 (s, 1H, Ar-His), 7.16–7.32 (m, 2H, Ar), 7.44 (d, *J* = 1.2 Hz, 1H, Ar-His), 7.46–7.54 (m, 3H, Ar), 7.57 (d, *J* = 1.3 Hz, 1H, CH-Py), 7.81–7.92 (m, 2H, *p*F-Ar), 7.93 (d, *J* = 1.3 Hz, 1H, CH-Py), 8.18–8.33 (m, 2H, Ar), 8.61 (d, *J* = 7.4 Hz, 1H, NH-His). ^13^C NMR (101 MHz, DMSO-*d*_6_): δ 28.76, 34.49, 52.44, 52.83, 116.14, 116.80, 119.18, 122.50, 128.16, 128.70, 129.35, 129.42, 129.91, 133.75, 135.28, 137.64, 147.09, 155.87, 157.47, 162.62 (d, *J*_C-F_ = 246.6 Hz), 168.36, 172.40. Anal. calcd. for C_26_H_23_FN_4_O_3_S C, 63.66; H, 4.73; N, 11.42; S, 6.54; found C, 63.68; H, 4.74; N, 11.40; S, 6.52.

### 2.2. Biology

*Compounds and peptide*. Ribavirin (RBV, 1-D-ribofuranosyl-1,2,4-triazole-3-carboxamide) and oseltamivir carboxylate (OSC) were purchased from Roche. Each test compound was dissolved in 100% DMSO. The PB1_(1–15)_–Tat peptide was synthesized and purified by the Peptide Facility of the CRIBI Biotechnology Center (University of Padua, Italy). This peptide corresponds to the first 15 amino acids of PB1 protein fused to a short sequence of HIV Tat protein (amino acids 47–59).

*Cells and Virus*. Madin-Darby canine kidney (MDCK) cells were grown in Dulbecco’s modified Eagle’s medium (DMEM, Life Technologies, Carlsbad, CA, USA) supplemented with 10% (*v*/*v*) fetal bovine serum (FBS, Life Technologies) and antibiotics (100 U/mL penicillin and 100 µg/mL streptomycin, Life Technologies). Cells were maintained at 37 °C in a humidified atmosphere with 5% CO_2_ and periodically tested for the absence of mycoplasma contamination. Influenza virus strain A/PR/8/34 (H1N1) was kindly provided by P. Digard (Roslin Institute, University of Edinburgh, United Kingdom).

*PA–PB1 interaction enzyme-linked immunosorbent assay (ELISA)*. The PA–PB1 interaction was detected by a procedure previously described [29,30]. Briefly, 96-well microtiter plates (Nuova Aptaca, Canelli, Italy) were coated with 400 ng of 6His-PA_(239–716)_ for 3 h at 37 °C and then blocked with 2% bovine serum albumin (BSA, Sigma, St. Louis, MO, USA) in PBS for 1 h at 37 °C. The 6His-PA_(239–716)_ protein was expressed in *E. coli* strain BL21(DE3)pLysS and purified as already described [20]. After washing, 200 ng of GST-PB1_(1–25)_, or GST alone as a control, was added in the absence or the presence of test compounds at various concentrations and incubated overnight at room temperature. *Escherichia coli*-expressed purified GST and GST-PB1_(1–25)_ proteins were obtained as previously described [20,31]. After washing, the interaction between 6His-PA_(239–716)_ and GST-PB1_(1–25)_ was detected with a horseradish peroxidase-coupled anti-GST monoclonal antibody (GenScript, Piscataway, NJ, USA) diluted 1:3000 in PBS supplemented with 2% FBS. Following washes, the substrate 3,3′,5,5′-tetramethylbenzidine (TMB, KPL) was added, and absorbance was measured at 450 nm by an ELISA plate reader (MultiSkan FC, ThermoFisher Scientific, Waltham, MA, USA). Values obtained from the samples treated with only DMSO were set as 100% of PA–PB1 interaction.

*Cytotoxicity assay*. The cytotoxicity of compounds was tested in MDCK cells using the 3-(4,5-dimethylthiazol-2-yl)-2,5-diphenyl tetrazolium bromide (MTT) method, as previously reported [32]. Briefly, MDCK cells (seeded at a density of 2 × 10^4^ per well) were grown in 96-well plates for 24 h and then treated with serial dilutions of test compounds, or DMSO as a control, in DMEM supplemented with 10% FBS. After incubation at 37 °C for 48 h, 5 mg/mL of MTT (Sigma, Tokyo, Japan) in PBS was added into each well and incubated at 37 °C for a further 4 h. Successively, a solubilization solution was added to lyse the cells and incubated overnight at 37 °C. Finally, optical density was read at the wavelength of 570 nm on a microtiter plate reader (MultiSkan FC, ThermoFisher Scientific).

*Plaque reduction assay (PRA)*. The antiviral activity of test compounds against the influenza A virus was tested by PRA, as previously described [33]. MDCK cells were seeded at 5 × 10^5^ cells/well into 12-well plates and incubated at 37 °C for 24 h. The following day, the culture medium was removed, and the monolayers were first washed with serum-free DMEM and then infected with the IAV A/PR/8/34 strain at 40 PFU/well in DMEM supplemented with 2 μg/mL of TPCK-treated trypsin (Worthington Biochemical Corporation, Freehold, NJ, USA) and 0.14% BSA, and incubated for 1 h at 37 °C. The influenza virus infection was performed in the presence of different concentrations of test compounds or a solvent (DMSO) as a control. After virus adsorption, DMEM containing 2 μg/mL of TPCK-treated trypsin, 0.14% BSA, 1.2% Avicel, and DMSO or test compounds was added to the cells. At 48 h post-infection, cells were fixed with 4% formaldehyde and stained with 0.1% toluidine blue. Viral plaques were counted, and the mean plaque number in the DMSO-treated control was set at 100%.

### 2.3. Molecular Modeling

The crystallographic structure of the PA–PB1 protein–protein complex coded by PDB-ID: 3CM8 was used as a rigid receptor in molecular docking simulations [26]. The PB1 peptide was removed to allow small molecules docking within the PB1-binding pocket on PA. Small molecules were designed and prepared for docking through ionization at pH 7.4 and energy minimization, using Fixpka version 2.0.0.3 (QUACPAC Application 2.0.0.3, OpenEye, Cadence Molecular Sciences, Santa Fe, NM, USA) [34] and Szybki version 1.10.0.3 with the MMFF94S force field (Szybki application 1.10.0.3, OpenEye, Cadence Molecular Sciences, Santa Fe, NM, USA), respectively (OpenEye QUACPAC 2.0.0.3.; OpenEye SZYBKI 1.10.0.3.) [35]. The molecular ionization of compounds bearing the imidazole ring was further confirmed by MoKa (Molecular Discovery, Hertfordshire, UK) [36,37,38]. In accordance with previous works [20,23,24,39], docking simulations were carried out with the GOLD docking program, version 2020.1 [40,41]. The GoldScore function was used for docking and scoring purposes. The binding site was centered on the N atom of Lys-643 and had a radius of 14 Å.

The ten top-ranking poses of each small molecule were stored and visually inspected. The maximum search efficiency (200%) of the docking algorithm was used, while all other parameters were kept at their default values.

## 3. Results and Discussion

### 3.1. Chemistry

**Scheme 1:**
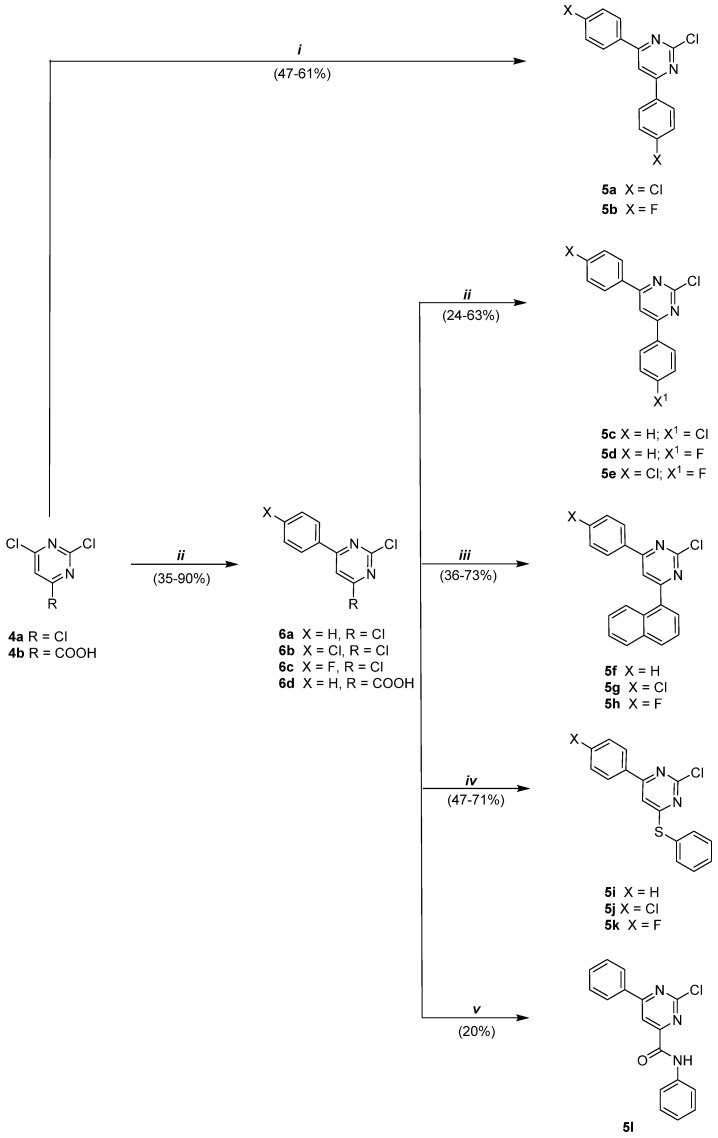
Synthesis of pyrimidine intermediates **5a**–**l**. **Reagents and conditions:** (i) opportune phenylboronic acid (2 eq), 1 M Na_2_CO_3_, PPh_3_, Pd(OAc)_2_, THF, N_2_ atm, reflux, 3 h (to obtain **5a**,**b**), 47–61%; (ii) opportune phenylboronic acid (1 eq), 1 M Na_2_CO_3_, PPh_3_, Pd(OAc)_2_, THF, N_2_ atm, reflux, 3–6 h (to obtain **6a**–**d**, 35–90% and **5c**–**e**, 24–63%); (iii) 1-naphtalenboronic acid, aq. sol Na_2_CO_3_, PPh_3_, Pd(OAc)_2_, DME, N_2_ atm, reflux, 3 h, 36–73%; (iv) Method A: thiophenol, H_2_O/Acetone, NaOH, rt, 3 h (to obtain **5i**, 71%); Method B: thiophenol, dry DMF, NaH, N_2_ atm, 0 °C to rt, 3 h (to obtain **5j**,**k**, 47–71%); (v) aniline, EDC, HOBt, DMF, N_2_ atm, rt, 3 h, 20%.

**Scheme 2:**
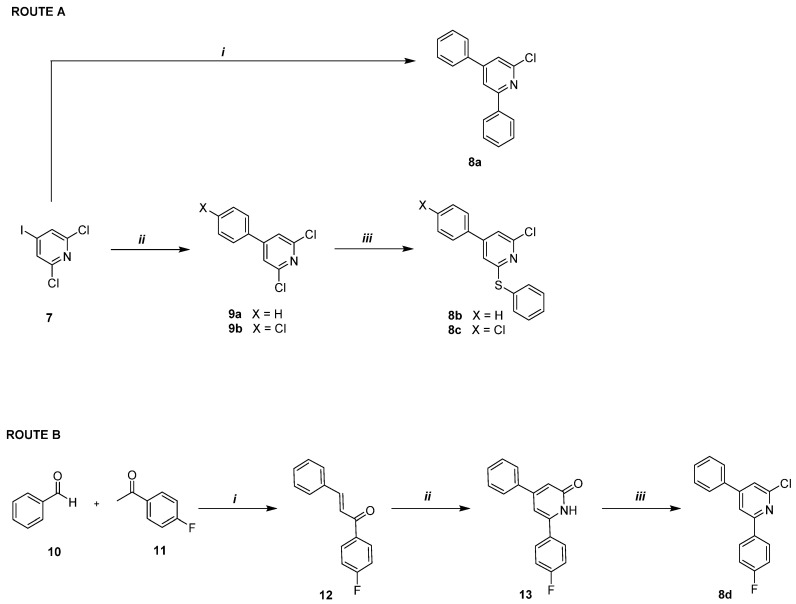
Synthesis of pyridine intermediates **8a**–**d**. **Reagents and conditions:** (**Route A**) (i) phenylboronic acid (2 eq), 1 M K_2_CO_3_, Pd(PPh_3_)_4_, DME, N_2_ atm, reflux, 16 h, 60%; (ii) opportune phenylboronic acid (1.1 eq), 1 M Na_2_CO_3_, PPh_3_, Pd(OAc)_2_, THF, N_2_ atm, reflux, 3 h, 73–74%; (iii) thiophenol, dry DMF, NaH, N_2_ atm, 0 °C 1 h, then rt, 3 h, 82–95%; (**Route B**) (i) 4 M NaOH, EtOH, rt, 3 h, 80%; (ii) ethyl 2-nitroacetate, NH_4_OAc, EtOH, N_2_ atm, reflux, 6 h, 25%; (iii) POCl_3_/DMF (1:1), CHCl_3_, reflux, 12 h, 60%.

Different synthetic routes were applied to obtain the desired compounds **2a**–**n** and **3a**–**n**. The first part of the chemical work was devoted to the synthesis of the key pyrimidine and pyridine intermediates **5a**–**l** and **8a**–**d**, respectively. Most of these derivatives were conveniently prepared by Suzuki–Miyaura cross-coupling reactions (Scheme 1 and Scheme 2 (Route A)).

Starting from the pyrimidine series, the commercially available 2,4,6-dichloropyrimidine (**4a**) was used as a common precursor for the synthesis of most intermediates (compounds **5a**–**l**) (Scheme 1). The 4,6-diphenylpyrimidine derivatives **5a**,**b** were obtained by one-step Suzuki reaction in the presence of two equivalents of the appropriate phenylboronic acid (47–61%). The intermediates **5c**–**l** were synthesized from the corresponding 4-phenylpyrimidines **6a**–**d**, easily obtained (35–90%) by a first Suzuki–Miyaura reaction on **4a**. In detail, the treatment of **6a**–**c** with the opportune organoboranes, under classical Suzuki conditions, regioselectively gave the 4,6-disubstituted pyrimidines **5c**–**h**, respectively, in good yields (24–73%). Alternatively, a nucleophilic aromatic substitution on substrates **6a**,**b** with thiophenol under basic conditions afforded the derivatives **5i**–**k** in satisfactory yields (47–71%). Finally, an amidation reaction of the carboxylic acid precursor **6c**, in the presence of EDC and HOBt as coupling agents, dry DIPEA, and the aniline in dry DMF, gave the intermediate **5l**.

Two synthetic approaches have been developed for obtaining the pyridine derivatives **8a**–**d**, as shown in Scheme 2 (Route A and B). The intermediates **8a**–**c** were synthesized through a Suzuki cross-coupling reaction. In detail, **8a** was obtained by reacting the 2,6-dichloro-4-iodopyridine **7** and phenyl boronic acid in the presence of catalysts, i.e., tetrakis and an aqueous solution of sodium carbonate, in DME. Alternatively, to afford compounds **8b**,**c** (82–95%), the products of the Suzuki cross-coupling reaction, i.e., **9a**,**b**, were used as electrophile species for an aromatic nucleophilic substitution in the presence of thiophenol (Route A). On the other hand, the intermediate **8d** was synthesized in good yield via a base-catalyzed Claisen–Schmidt condensation between *p*-F-acetophenone **10** and benzaldehyde **11**, as shown in Route B. The corresponding chalcone derivative **12** was reacted via a base-catalyzed Michael addition with ethyl 2-nitroacetate, leading to the functionalized pyridin-2(1*H*)-one **13**. Derivative **13** was chlorinated using the Vilsmeier complex (POCl_3_/DMF, 1:1) in anhydrous chloroform at reflux conditions, affording the key intermediate **8d**.

A specific rationale drove the synthesis of compounds of the series **2** and **3**. We decided to prepare compounds **2a**–**n** to explore the two hydrophobic areas II and III of the PA subunit (**1a**) by means of different aromatic groups on the C4 and C6 positions of the heterocyclic ring. In particular, we chose the unsubstituted phenyl group and the *p*-halogen substituted phenyl group to investigate the SAR of a range of substituents and the 1-naphthyl group to extend the molecule’s hydrophobic interactions. Furthermore, we inserted the thiophenyl moiety to evaluate the effect of a more flexible aromatic substituent on the activity. Finally, we introduced an aromatic amide to explore possible new interactions with the PA key residues.

**Scheme 3:**
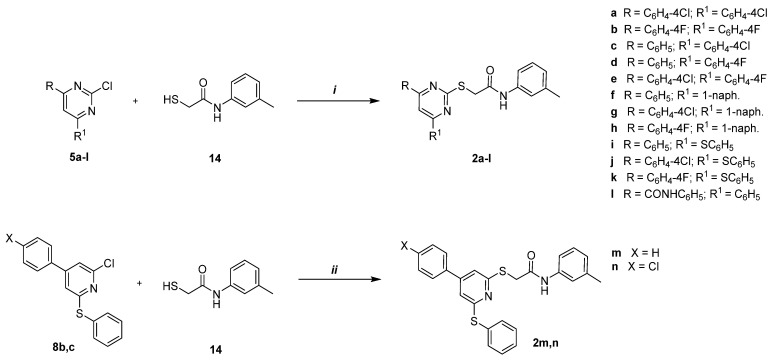
The final step for the synthesis of the pyrimidine and pyridine derivatives **2a**–**n**. **Reagents and conditions:** (i) dry K_2_CO_3_, dry ACN, N_2_ atm, rt, 5 h, 20–62%; (ii) dry K_2_CO_3_, CuI, L-proline, dry DME, N_2_ atm, reflux, 24 h, 20%.

The final products **2a**–**l** (20–62%) were synthesized by aromatic nucleophilic substitution on the appropriate aromatic electrophile **5a**–**l** with the nucleophile 2-mercapto-*N*-(m-tolyl)acetamide **14** in acetonitrile, as shown in Scheme 3. The different electron-deficient nature of the pyrimidine ring required stronger aromatic nucleophilic substitution conditions for obtaining compounds **2m**,**n**. In detail, the CuI/L-proline-catalyzed system allowed the substitution at C2 of intermediates **8b**,**c** with the mercapto-*N*-(*m*-tolyl)acetamide side chain (Scheme 3).

Compounds **3a**–**n** were designed using a mix and match approach, combining the pyrimidine or pyridine ring scaffold with the most promising amino acids of the first generation of cyano-pyridine [23]. Since the biological results of the derivatives **2a**–**n** were better for derivatives endowed with small aromatic groups, we prepared compounds bearing unsubstituted phenyl groups or *p*-fluoro-substituted phenyl groups at C4 and C6.

**Scheme 4:**
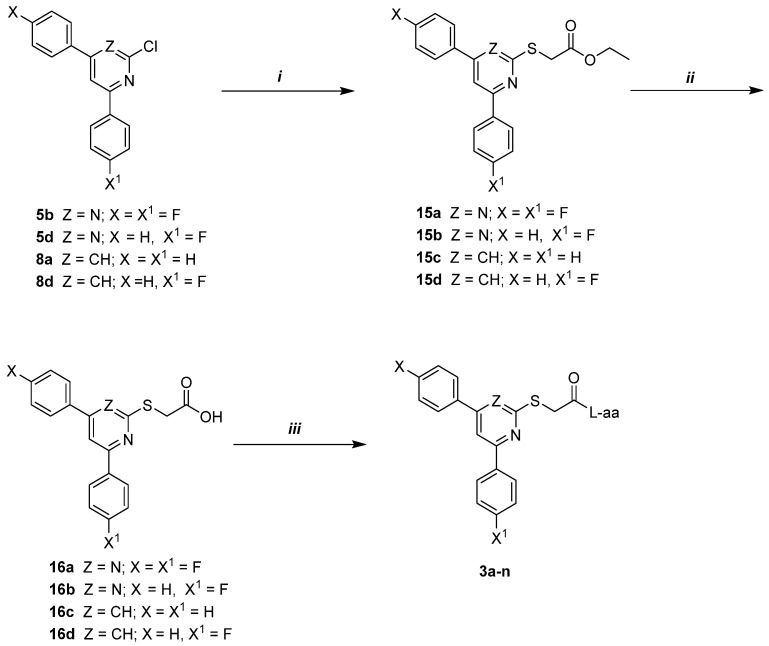
Final steps for the synthesis of the pyrimidine and pyridine derivatives **3a**–**n**. **Reagents and conditions:** (i) Method A: ethyl-thioglycolate, Cs_2_CO_3_, dry ACN, 30–40 °C, 1 h (to obtain **15a**,**b**, 68–76%); Method B: ethyl-thioglycolate, Cs_2_CO_3_, dry DMF, 100 °C, 6 h (to obtain **15c**,**d**, 25–40%); (ii) aq. sol. NaOH 5% *w*/*v*, THF, rt, 16 h; (iii) opportune L-aa-methyl ester, EDC, HOBt, dry DIPEA, dry DMF, N_2_ atm, rt, 4–6 h, 20–64%.

A different synthetic approach was required to afford the hybrid compounds **3a**–**n**. Diarylated-pyrimidine/pyridines intermediates, i.e., **5b**,**d** and **8a**,**b**, were reacted by aromatic nucleophilic substitution with ethyl thioglycolate, affording intermediates **15a**–**d** (25–76%) (Scheme 4). Reaction conditions differed according to the reactant properties, and in the pyridine series, temperature control was essential to avoid forming a highly fluorescent disulfide side product. The C2-substituted intermediates **15a**–**d** underwent a saponification reaction, affording the corresponding carboxylic derivatives **16a**–**d**, which were reacted in the last amidation step to synthesize the final compounds **3a**–**n** (20–64%).

### 3.2. Biology

Compounds **2a**–**n** and **3a**–**n** were evaluated for their antiviral activity and tested as PA–PB1 PPI inhibitors. In detail, an ELISA-based assay on the PA–PB1 complex, PRA in MDCK cells infected with IAV A/PR/8/34 (H1N1) strain, and a cytotoxicity analysis in the MDCK cell line by MTT assays were performed (Table 1).

The PB1_(1–15)_-Tat peptide [42] was used as a positive control for inhibition in the ELISA, while ribavirin (RBV) [43] and oseltamivir (OSC) [44] were used as positive controls in the PRA. RBV is a viral polymerases inhibitor, although it does not act as an RdRp disruptor, while OSC is an NA inhibitor and a first-choice drug in the therapy of influenza.

A SAR analysis of pyrimidine derivatives **2a**–**l** revealed that compounds bearing two phenyl groups directly attached to the heterocyclic core at the C4 and C6 position (compounds **2a**–**e**) were endowed with better activity in ELISA compared to naphthyl (**2f**–**h**) and thiophenyl (**2i**–**k**) derivatives, with **2i** being the exception and proving the most effective of this series in ELISA assay (IC_50_ = 28.8 ± 1.5 µM). This activity profile can likely be attributed to the better accommodation of the phenyl ring in the two hydrophobic areas II and III of the PA subunit than the bulkier naphthyl group or the more flexible thiophenyl chain.

An in-depth SAR analysis at position C6 showed that monosubstituted compounds **2c** and **2d** displayed the highest activity in the series **2a**–**e**. Specifically, compounds **2c** and **2d** showed IC_50_ values in ELISA of 86.0 and 90.1 μM, respectively, EC_50_ in the PRA assay of 45.5 and 2.8 μM, and a good cytotoxicity profile (CC_50_ > 250 μM). Notably, **2d** represents a significant advancement compared to RdRp inhibitors previously synthesized by our group, possessing a better or the same order of magnitude IC_50_ value in ELISA and enhanced antiviral activity. Moreover, **2d** is almost five-fold more active than RBV as an antiviral agent.

Among naphthyl derivatives, compounds **2f** and **2h**, despite their lack of activity as PA–PB1 disruptors, exhibited micromolar antiviral activity (EC_50_ 21.1 μM and 14.8 μM, respectively), possibly linked to a different mechanism of action.

Finally, thiophenyl derivatives **2i**–**k** and the amidic derivative **2l** showed an interesting antiviral profile (EC_50_ 4.6–32.9 μM), but this efficacy could be linked to PA–PB1 PPI inhibition only for compound **2i** (IC_50_ = 28.8 uM), whereas compounds **2j**, **2k**, and **2l** were characterized by high cytotoxicity; thus, for these compounds, the observed activities in cell-based assays are likely due to toxicity, rather than real antiviral activity.

On the other hand, the pyridine derivatives **2m**,**n**, featuring a thiophenyl chain, did not exhibit significant toxicity (CC_50_ > 250 μM), in contrast to what was observed with the pyrimidine analogs **2i** and **2j** (CC_50_ 73.3 and 16.0 μM, respectively). In addition, compound **2n**, bearing a 4-chloro phenyl chain on the C4 position of the pyridine core, showed IC_50_ = 87.0 μM, EC_50_ = 62.9 μM, and CC_50_ > 250 μM, emerging as a promising PA–PB1 disruptor.

These results prompted us to decorate the most promising pyridine and pyrimidine derivatives with amino acidic moieties to try affording bioactivity profiles such as those obtained in our previous study [23,24]. In particular, we selected isoleucine, which led to the strongest compound **1b** (Figure 2), valine, tyrosine, and histidine, whose respective compounds showed good cytotoxic profiles and IC_50_ values of 167, 18, and 32 μM, respectively, and EC_50_ values of 52, >100, and 80 μM, respectively [23]. Based on this rationale, compounds **3a**–**n** were synthesized. Unfortunately, they did not yield the expected PA–PB1 PPI inhibition or antiviral activity, with the exception of **3d**, which exhibited modest PA–PB1 PPI inhibition (IC_50_ = 70.0 μM), albeit associated with cytotoxicity.

### 3.3. Molecular Modeling

A molecular modeling study was performed to shed light on the activity profile of compounds of series **2** and **3**. Specifically, compounds **2d**, **2i**, **2n**, **3a**, **3d**, and **3n** were selected for this study, based on their peculiar bioactivity profiles. Indeed, **2d** was the pyrimidine derivative that exerted the strongest antiviral effect, **2i** proved the most effective in PA–PB1 PPI inhibition by ELISA assay, **2n** was the most effective compound of the pyridine series, and **3d** was the most potent in PA–PB1 PPI inhibition through ELISA assay among the series **3**, e.g., amino acid-derived compounds. Additionally, **3a** and **3n** were valuable examples of inactive molecules bearing high chemical similarity to bioactive hits.

**Figure 3:**
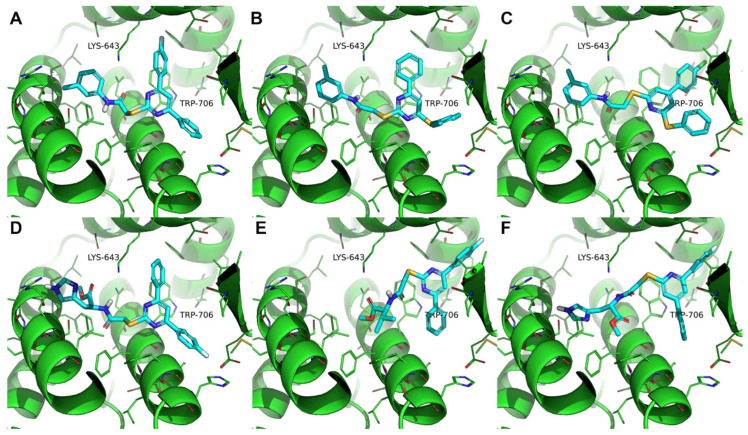
Docking-based binding mode of bioactive inhibitors **2d** (**A**), **2i** (**B**), **2n** (**C**), **3d** (**D**), and inactive molecules **3a** (**E**) and **3n** (**F**), towards the crystallographic structure of PA–PB1 PPI complex coded by PDB-ID: 3CM8. PB1 coordinates were removed in docking simulations. PA is shown as green cartoon coils and lines. Residues within 5 Å from the ligands are shown as lines. Two key residues, Lys-643 and Trp-706, that are discussed in the text are labeled. Residues numbering is taken from the crystallographic structure 3CM8. Small molecules are shown as cyan sticks, non-polar H atoms are omitted.

Molecular docking simulations were carried out with the GOLD program [40,41], using the X-ray structure of PA in complex with PB1 as a rigid receptor (PDB-ID: 3CM8) [26]. To this aim, PB1 was removed from the crystallographic structure, and the selected small molecules were then docked into a ligand-binding conformation of PA. Docking results highlighted that removing the nitrile group from the pyridine ring, which had been designed to interact with Lys-643 [23,24], induced a switch in sub-pockets occupancy by aromatic substituents. Moreover, the lack of the anchor point on Lys-643 facilitated the stack of aromatic rings to the side chain of Trp-706 (Figure 3). As such, the thio-*N*-(*m*-tolyl)acetamide or the amino acidic side chain occupied sub-pocket III instead of sub-pocket I as in the previous design strategy (Figure 2). While this change was apparently not relevant in bioactive hits (**2d**, **2i**, **2n**, and **3d**, Figure 3A–D) that were able to fit the sub-pockets with a satisfactory matching, it proved detrimental for **3a** and **3n** (Figure 3E,F).

By comparing docking poses of bioactive vs. inactive compounds, we reasoned that in the case of **3a**, the lipophilic valine side chain did not fit with the polar edge of the helix where, for instance, a histidine side chain is well accommodated (**3d**) and is switched into a more lipophilic region (Figure 3D,E). Moreover, the pyridine ring of **3n** seemed to be captured by Trp-706 with higher efficacy than the pyrimidine ring in **3d**, which caused a shift of the docking pose with the ultimate positioning of the histidine ring of **3n** in a non-favorable lipophilic region (Figure 3D,F). Molecular docking also suggested that the introduction of flexibility through the thiophenyl group did not impair binding to the PA subunit, as in the case of **2i** and **2n** (Figure 3B,C).

Overall, molecular modeling shed light on the possible binding mode of representative members of series **2** and **3**, and also provided a structural rationale to justify the bioactivity data described in Table 1. Since subtle structural modifications may produce drastic changes in the bioactivity profiles of these molecules, additional generation of pyridine/pyrimidine-based inhibitors of PA–PB1 PPI with antiviral effects will be pursued.

## 4. Conclusions

In this study, based on the promising antiviral activity shown by our 3-cyano-4,6-diphenyl-pyridines with 2-thio-*N*-(*m*-tolyl)acetamide substituted [24,25], we rationally designed a series of structurally analogous pyrimidine and pyridine compounds **2a**–**n.** Among the latter, **2d**, possessing a pyrimidine core and a phenyl and 4-chloro phenyl ring at the C4 and C6 positions, respectively, emerged as a significant breakthrough in the pursuit of new PPI inhibitors, showing an IC_50_ value of 90.1 μM in PA–PB1 ELISA, an EC_50_ value of 2.8 μM in PRA, and a favorable cytotoxic profile.

These exciting findings prompted us to apply a mix and match approach. In detail, we replaced the thio-*N*-(*m*-tolyl)acetamidic side chain of the most active pyridine and pyrimidine derivatives of series **2** with the amino acidic chains that afforded the strongest compounds (e.g., **1b**) in our previous study [23]. This rational design led to the generation of the family of compounds **3a**–**n**. Unfortunately, these derivatives did not lead to the expected increase in activity, being generally inactive as PA–PB1 disruptors and antiviral agents, except **3d**, which showed IC_50_ = 70.0 µM. We performed a molecular modeling study to gain insights into this unexpected behaviour, showing that the lack of the nitrile group determined a change in sub-pocket occupancy in the PB1 binding site of PA. Docking poses of bioactive and inactive derivatives proved useful to structurally encode the bioactivity profiles and might guide the design of additional generations of PA–PB1 PPI inhibitors.

Overall, this study led us to perform a comprehensive SAR analysis into this class of compounds, to obtain a deep insight into their mechanism of action via in silico study, and to identify a promising lead compound (**2d**) active as RdRp inhibitor and antiviral agent, thereby providing a contribution to a field which still seeks new efficacious therapies.

## Figures and Tables

**Table 1:** Structure and biological activity of new compounds **2a**–**n** and **3a**–**n**.

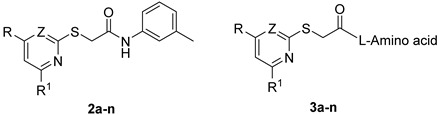							
Cpd	Z	R	R^1^	L-Amino Acid	IC_50_ (ELISA PA–PB1) [µM] ^a^	EC_50_ (PRA) [µM] ^b^	CC_50_ (MDCK Cells) [µM] ^c^
**1a** [25]	CCN	C_6_H_4_-4CH_3_	C_6_H_5_	-	80	ND ^d^	ND ^d^
**1b** [23]	CCN	C_6_H_4_-4CH_3_	C_6_H_5_	Ile-OCH_3_	36 ± 3	39 ± 2	>250
**1c** [24]	N	C_6_H_4_-4CF_3_	C_6_H_4_-4CF_3_	-	>200	26.5 ± 4.3	>250
**1d** [24]	N	C_6_H_5_	C_6_H_5_	-	165 ± 18	3.5 ± 0.5	10.1 ± 1.7
**1e** [24]	CH	C_6_H_5_	C_6_H_5_	-	52.6 ± 9.0	7.3 ± 1.3	>250
**2a**	N	C_6_H_4_-4Cl	C_6_H_4_-4Cl	-	70.8 ± 19.5	>100	>250
**2b**	N	C_6_H_4_-4F	C_6_H_4_-4F	-	137 ± 35	62.5 ± 4.3	>250
**2c**	N	C_6_H_5_	C_6_H_4_-4Cl	-	86.0 ± 5.3	45.5 ± 2.1	>250
**2d**	N	C_6_H_5_	C_6_H_4_-4F	-	90.1 ± 30.2	2.8 ± 0.6	>250
**2e**	N	C_6_H_4_-4Cl	C_6_H_4_-4F	-	125 ± 37	29.5 ± 0.7	206 ± 12
**2f**	N	C_6_H_5_	1-naph	-	>200	21.1 ± 8.0	>250
**2g**	N	C_6_H_4_-4Cl	1-naph	-	>200	>100	>250
**2h**	N	C_6_H_4_-4F	1-naph	-	>200	14.8 ± 1.6	>250
**2i**	N	C_6_H_5_	SC_6_H_5_	-	28.8 ± 1.5	4.6 ± 0.3	73.3 ± 9.1
**2j**	N	C_6_H_4_-4Cl	SC_6_H_5_	-	>200	32.9 ± 4.1	16.0 ± 4.2
**2k**	N	C_6_H_4_-4F	SC_6_H_5_	-	159 ± 29	16.4 ± 1.9	48.2 ± 2.5
**2l**	N	CONHC_6_H_5_	C_6_H_5_	-	125 ± 37	13.0 ± 5.5	46.3 ± 10.4
**2m**	CH	C_6_H_5_	SC_6_H_5_	-	141 ± 44	32.3 ± 3.2	>250
**2n**	CH	C_6_H_4_-4Cl	SC_6_H_5_	-	87.0 ± 15.7	62.9 ± 13.2	>250
**3a**	N	C_6_H_5_	C_6_H_4_-4F	Val-OCH_3_	>200	>100	234 ± 19
**3b**	N	C_6_H_5_	C_6_H_4_-4F	Ile-OCH_3_	>200	>100	>250
**3c**	N	C_6_H_5_	C_6_H_4_-4F	Tyr-OCH_3_	172 ± 7	>100	>250
**3d**	N	C_6_H_5_	C_6_H_4_-4F	His-OCH_3_	70.0 ± 2.8	>100	98.0 ± 11.3
**3e**	N	C_6_H_4_-4F	C_6_H_4_-4F	Val-OCH_3_	>200	>100	>250
**3f**	N	C_6_H_4_-4F	C_6_H_4_-4F	Ile-OCH_3_	>200	>100	>250
**3g**	N	C_6_H_4_-4F	C_6_H_4_-4F	Tyr-OCH_3_	>200	>100	>250
**3h**	N	C_6_H_4_-4F	C_6_H_4_-4F	His-OCH_3_	108 ± 14	>100	>250
**3i**	CH	C_6_H_5_	C_6_H_5_	Val-OCH_3_	>200	ND ^d^	ND ^d^
**3j**	CH	C_6_H_5_	C_6_H_5_	Ile-OCH_3_	>200	ND ^d^	ND ^d^
**3k**	CH	C_6_H_5_	C_6_H_5_	Tyr-OCH_3_	>200	ND ^d^	ND ^d^
**3l**	CH	C_6_H_5_	C_6_H_4_-4F	Ile-OCH_3_	>200	ND ^d^	ND ^d^
**3m**	CH	C_6_H_5_	C_6_H_4_-4F	Tyr-OCH_3_	>200	ND ^d^	ND ^d^
**3n**	CH	C_6_H_5_	C_6_H_4_-4F	His-OCH_3_	>200	ND ^d^	ND ^d^
PB1_(1–15)_-Tat peptide					31.7 ± 10.8	49.7 ± 5.1	>250
OSC					-	0.04 ± 0.01	>250
RBV					-	12.8 ± 3.2	>250

^a^ Activity of the compounds in the ELISA PA–PB1 interaction assay. Values represent the compound concentration (in µM) that reduced the interaction between PA and PB1 by 50% (IC_50_). ^b^ Antiviral activity of the compounds in plaque reduction assays against Flu A/PR/8/34 strain. Values represent the compound concentration (in µM) that inhibited 50% of plaque formation (EC_50_). ^c^ Cytotoxicity of the compounds exhibited in MTT assays. Values represent the compound concentration (in µM) that caused a 50% decrease in cell viability (CC_50_). The CC_50_ was assessed in MDCK cells. ^d^ ND = not determined. Reference compounds are the previously described compounds **1a**–**e**, PB1_(1–15)_-Tat peptide, oseltamivir carboxylate (OSC), and ribavirin (RBV).

## Data Availability

Data is contained within the article or Supplementary Material.

## Supplementary Materials

The following supporting information can be downloaded at: https://www.mdpi.com/article/10.3390/pharmaceutics16070954/s1, ^1^H-NMR and ^13^C-NMR of representative final compounds **2d**, **2e**, **2h**, **2i**, **2n**, **3a**, **3c**, **3j**, **3k**.
