# Supplementary material for: Exploring a New Generation of Pyrimidine and Pyridine Derivatives as Anti-Influenza Agents Targeting the Polymerase PA–PB1 Subunits Interaction
Source: Pharmaceutics. 2024 Jul 18;16(7):954. doi: 10.3390/pharmaceutics16070954 (PMC11279468; doi:10.3390/pharmaceutics16070954)
Supplement: Supplementary file 1 [file pharmaceutics-16-00954-s001.zip › pharmaceutics-3070989-supplementary.pdf]

Supplementary data

Exploring a new generation of pyrimidine and pyridine derivatives as anti-influenza agents targeting the polymerase PA-PB1 subunits interaction

Ilaria Giacchello,[1,#] Annarita Cianciusi,[1,#] Chiara Bertagnin,[2,#] Anna Bonomini,[2] Valeria Francesconi,[1] Mattia Mori,[3] Anna Carbone,[1,*] Francesca Musumeci,[1,*] Arianna Loregian,[2,§] Silvia Schenone[1,§]

[1] Department of Pharmacy, University of Genoa, Viale Benedetto XV 3, 16132 Genoa, Italy

[2] Department of Molecular Medicine, University of Padua, Via A. Gabelli 63, 35121 Padua, Italy

[3] Department of Biotechnology, Chemistry and Pharmacy, University of Siena, Via Aldo Moro 2, 53100 Siena, Italy

* Correspondence: anna.carbone1@unige.it; francesca.musumeci@unige.it
# These Authors equally contributed to the work
§ Co-last Authors

Table of Contents:

- ^{1}H-NMR and ^{13}C-NMR of representative final compounds **2d, 2e, 2h, 2i, 2n, 3a, 3c, 3j, 3k.**

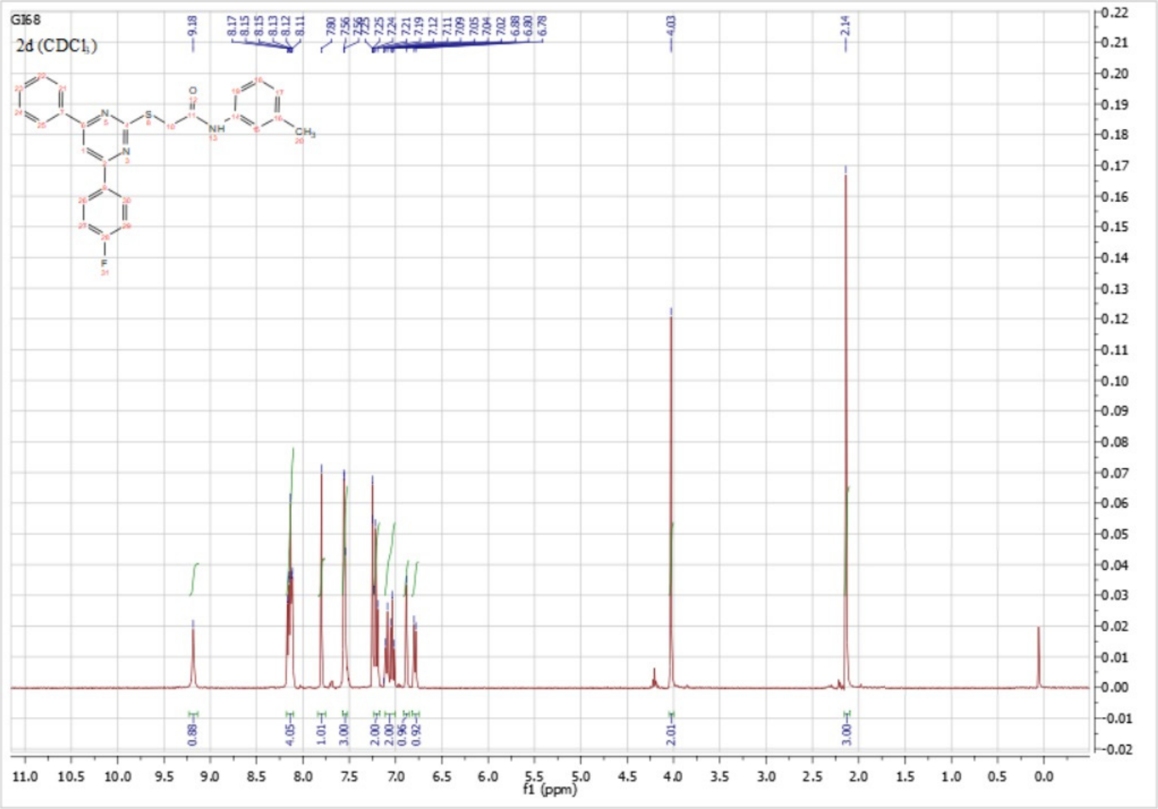

GI68
2d (CDCl3)
9.18
8.17 8.15 8.15 8.13 8.12 8.11
7.80 7.56 7.56 7.55 7.25 7.24 7.21 7.19 7.12 7.11 7.09 7.05 7.04 7.02 6.88 6.80 6.78
4.03
2.14
0.88
4.05
1.01
3.00
2.00
2.00
0.96
0.92
2.01
3.00
f1 (ppm)

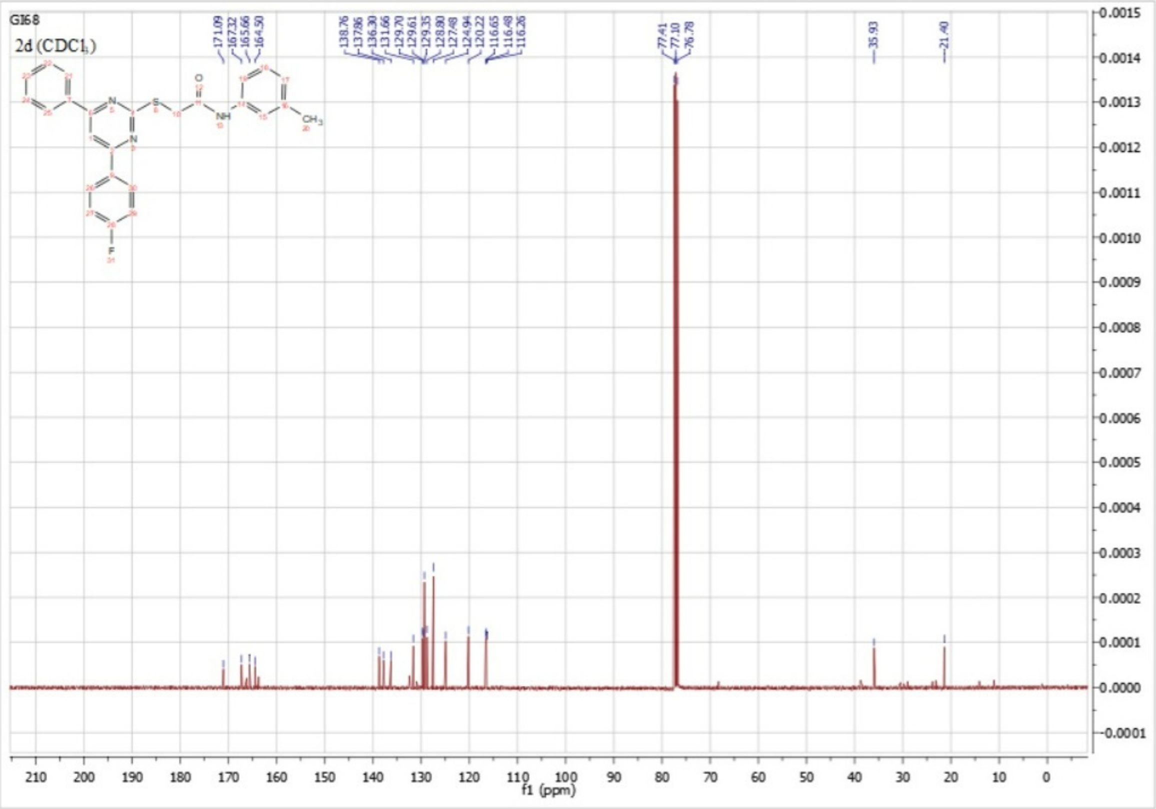

GI68
2d (CDCl3)
171.09 167.32 165.66 164.50
138.76 137.86 136.30 131.66 129.70 129.61 129.35 128.80 127.48 124.94 120.22 116.65 116.48 116.26
77.41 77.10 76.78
35.93
21.40
f1 (ppm)

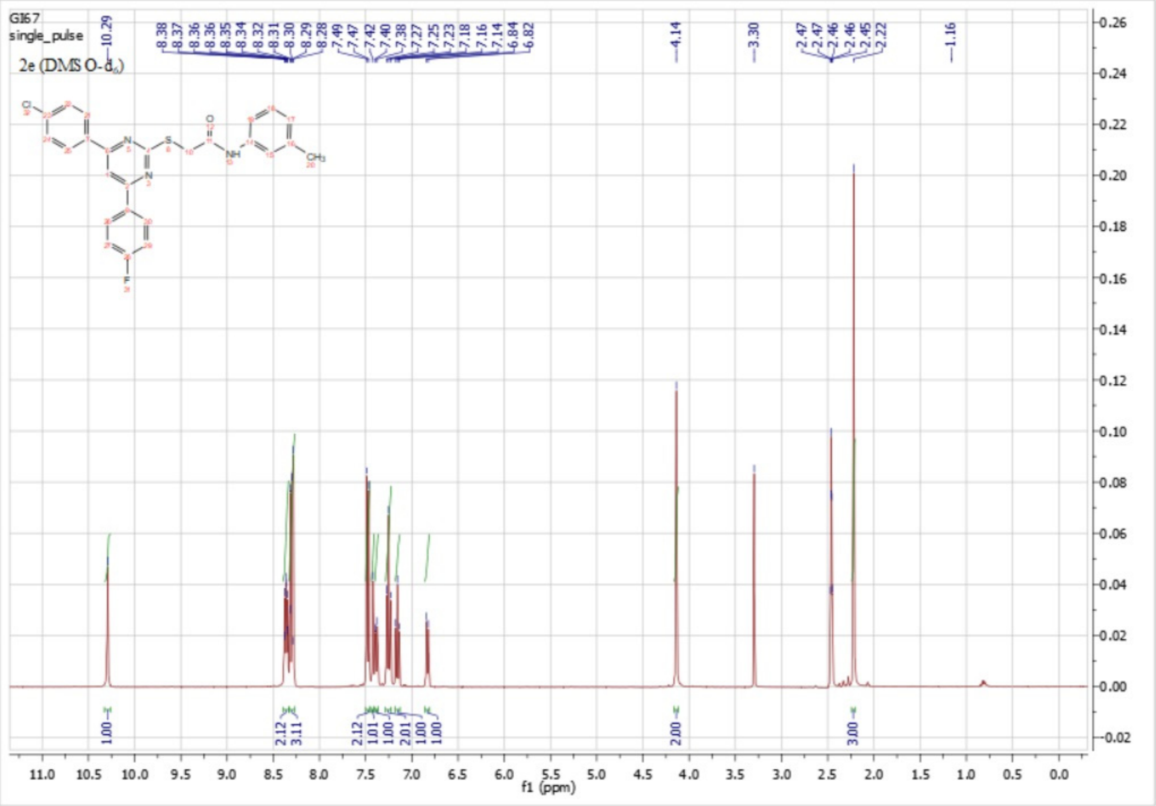
GI67
single_pulse
2e (DMSO-d6)
f1 (ppm)

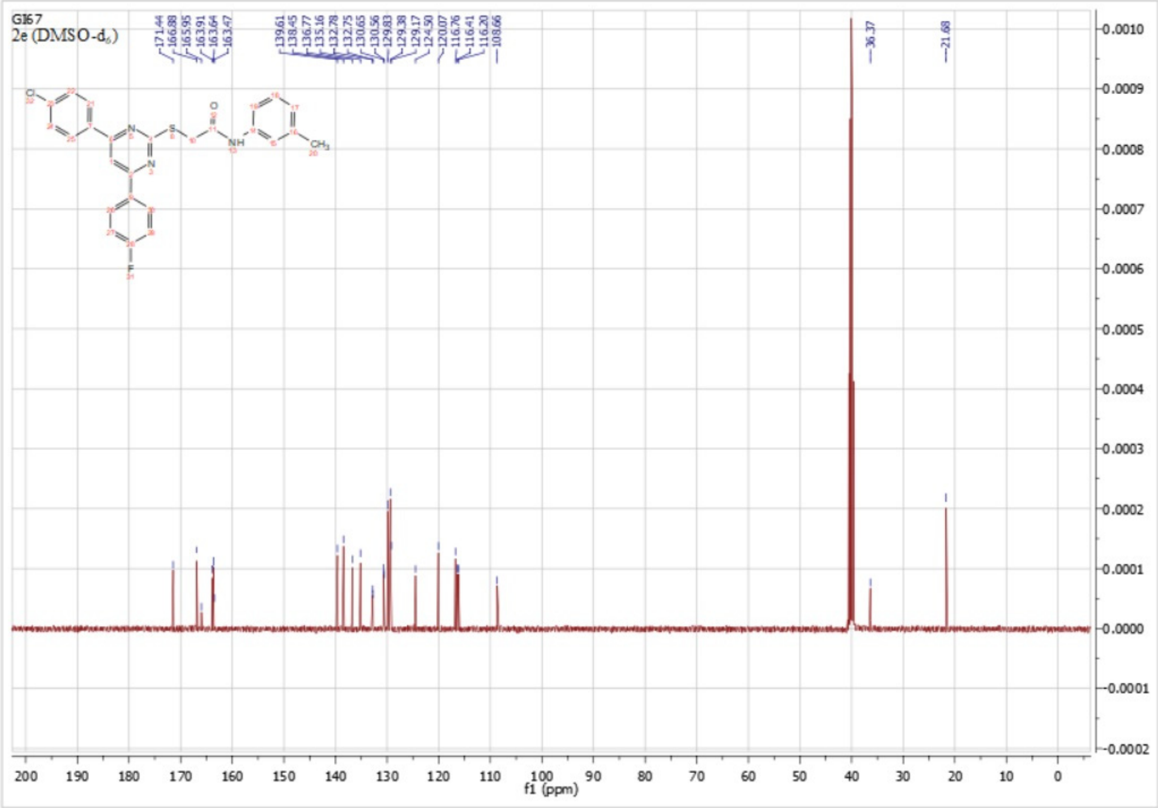
GI67
2e (DMSO-d6)
f1 (ppm)

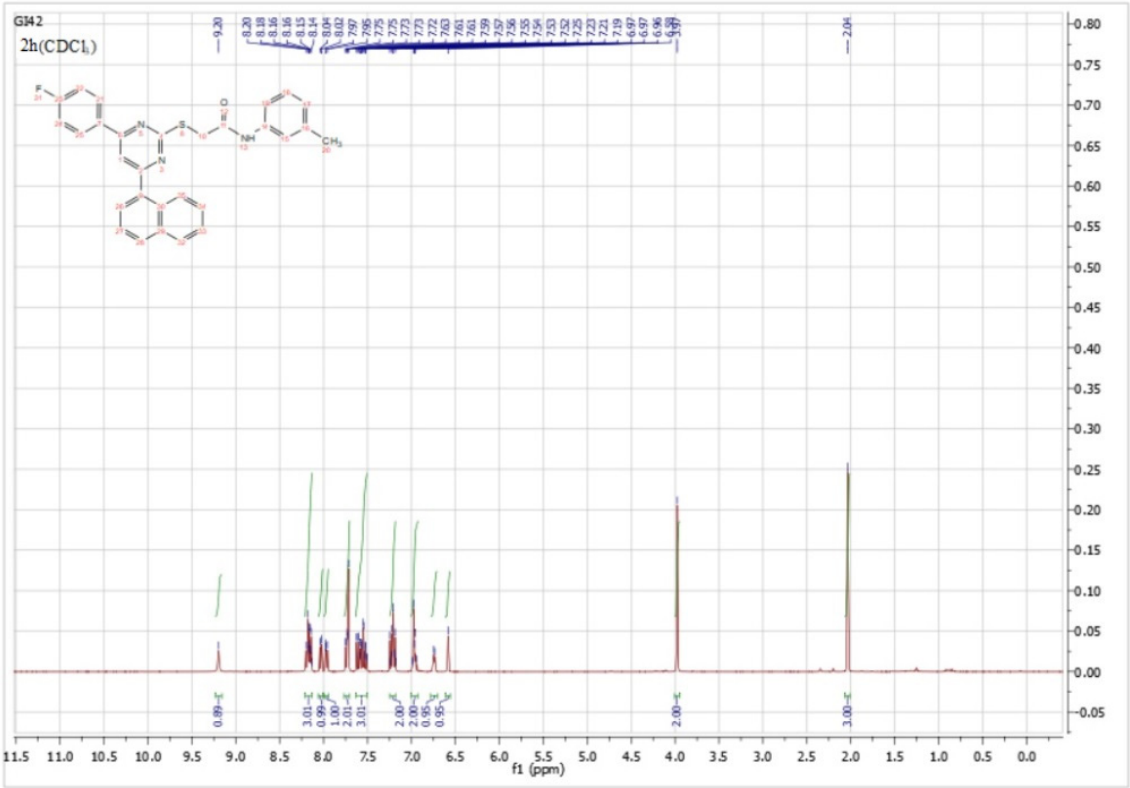
GI42
2h(CDCl3)

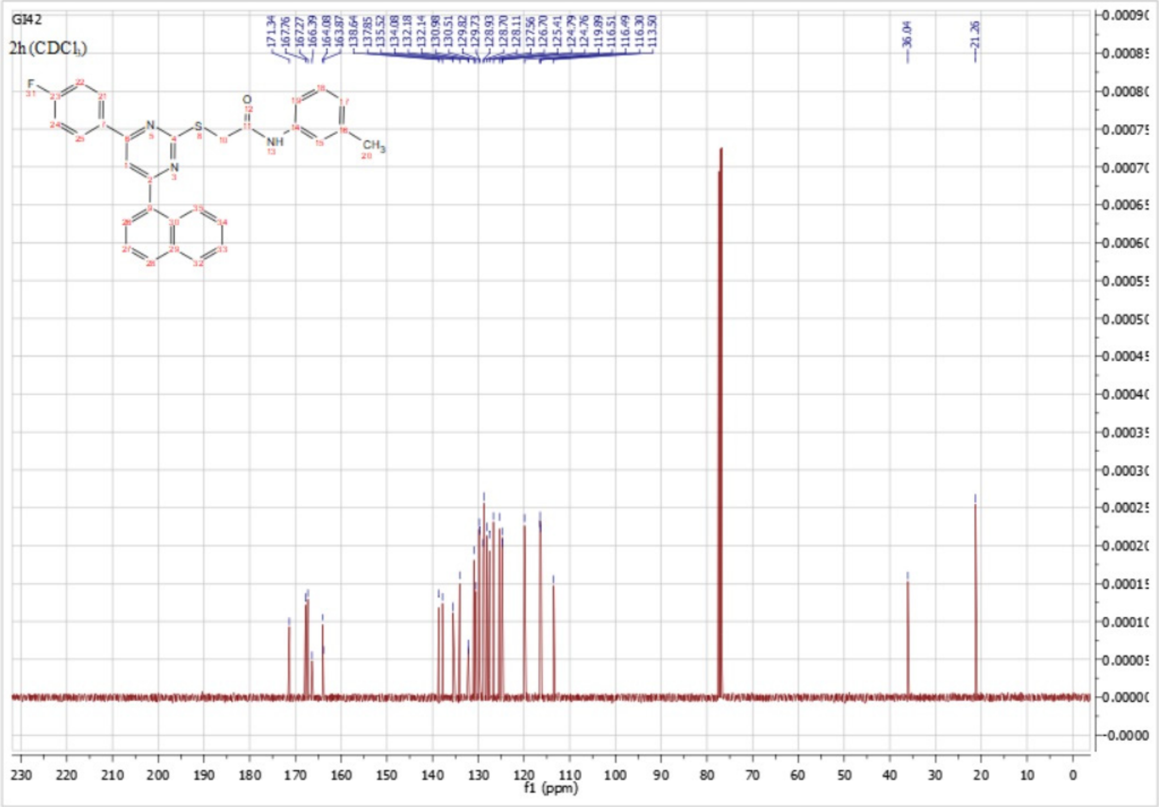
GI42
2h(CDCl3)

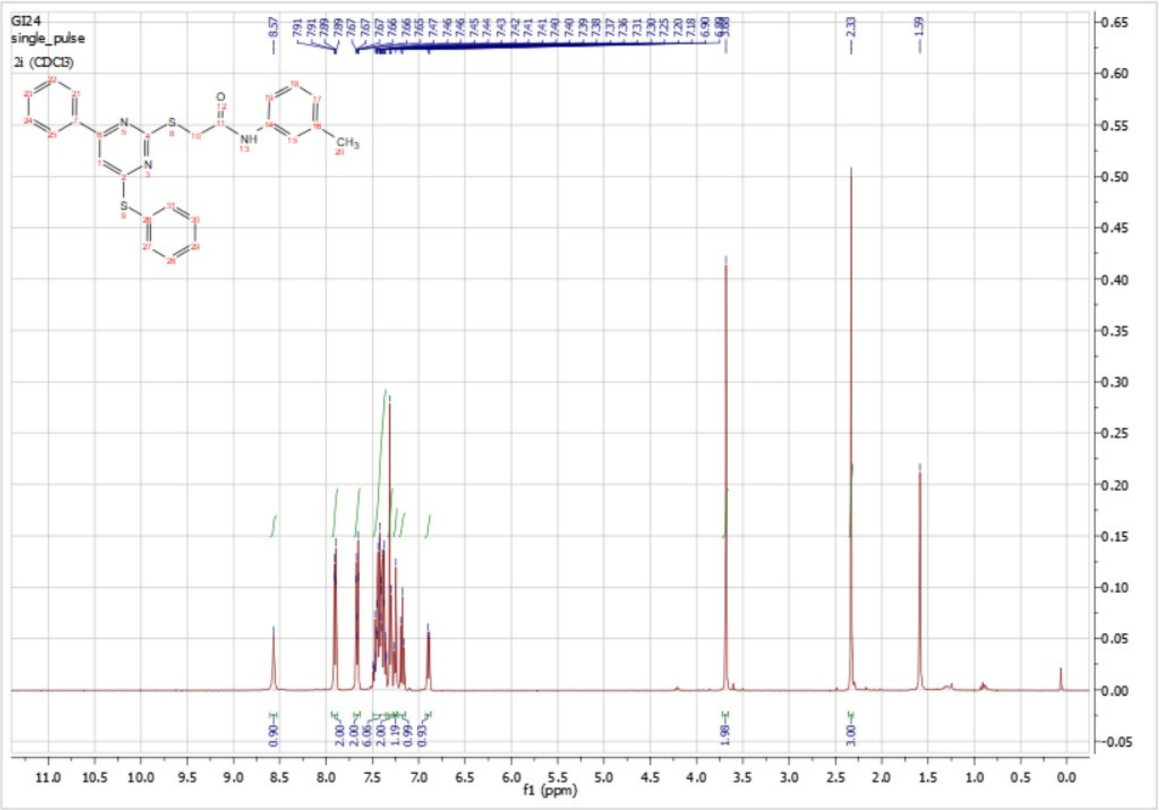
GI24
single_pulse
2i (CDCl3)
f1 (ppm)

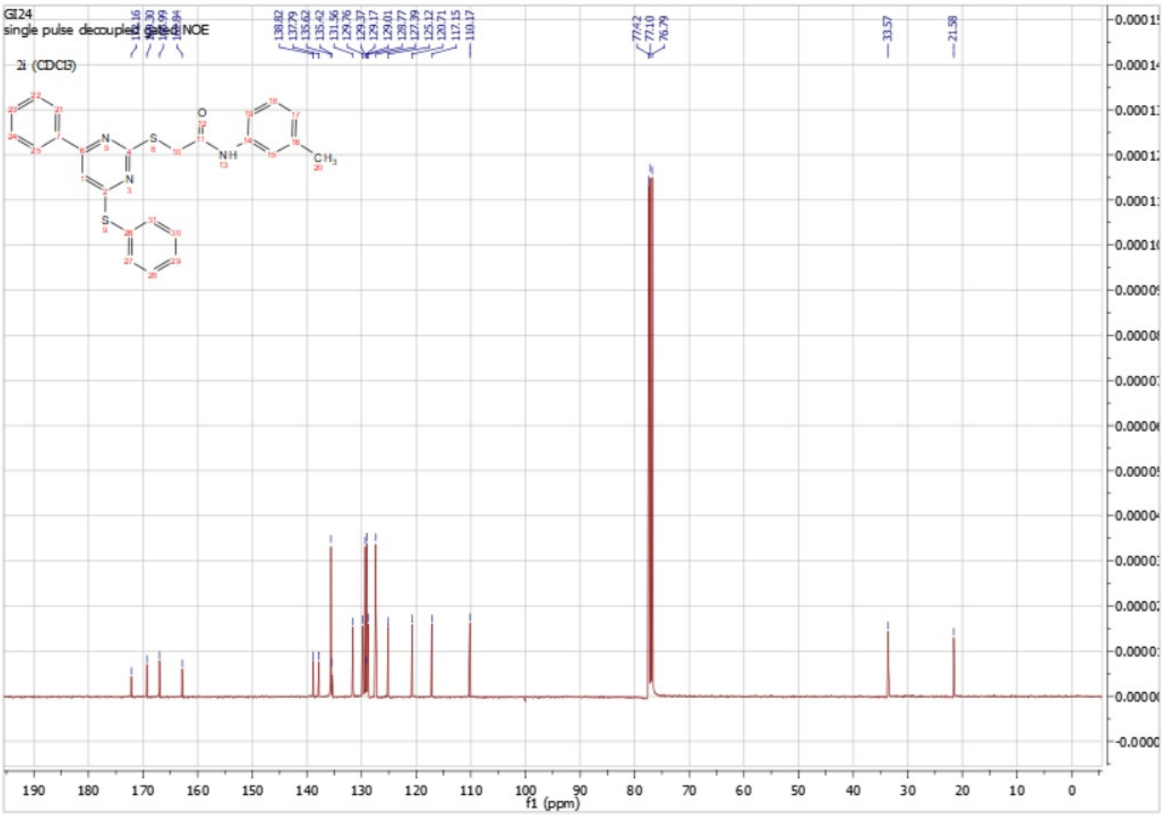
GI24
single pulse decoupled gated NOE
2i (CDCl3)
f1 (ppm)

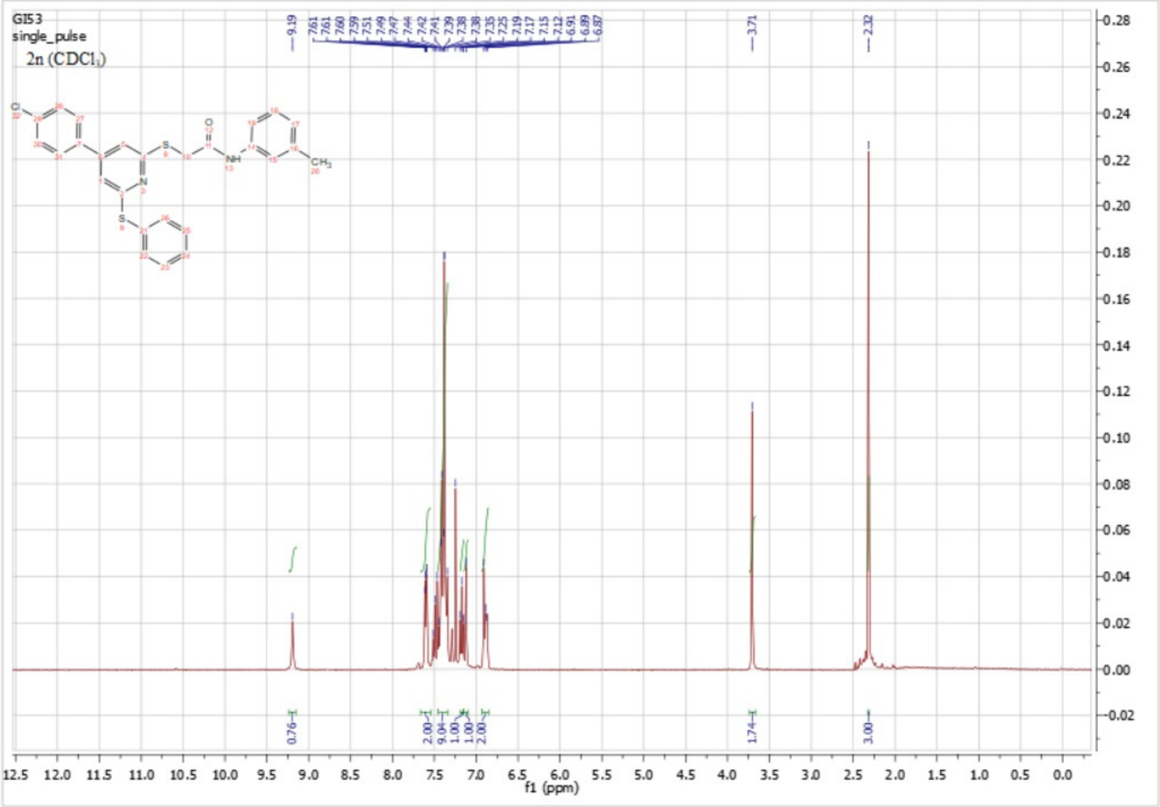
GI53
single_pulse
2n (CDCl3)
9.19
7.61 7.61 7.60 7.59 7.51 7.49 7.47 7.44 7.42 7.41 7.39 7.38 7.38 7.35 7.25 7.19 7.17 7.15 7.12 6.91 6.89 6.87
3.71
2.32
0.76
2.00 9.04 1.00 1.00 2.00
1.74
3.00
f1 (ppm)

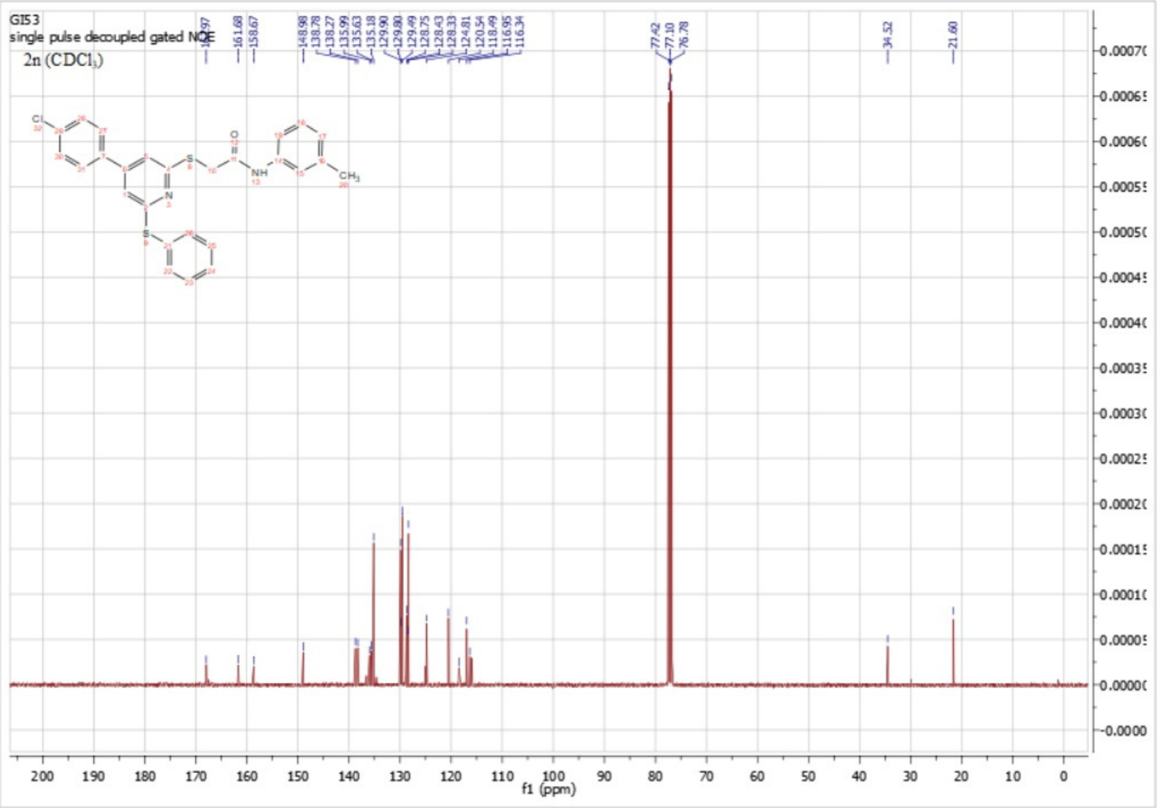
GI53
single pulse decoupled gated NOE
2n (CDCl3)
161.68
158.67
148.98 138.78 138.27 135.99 135.63 135.18 129.90 129.80 129.49 128.75 128.43 128.33 124.81 120.54 118.49 116.95 116.34
77.42 77.10 76.78
34.52
21.60
f1 (ppm)

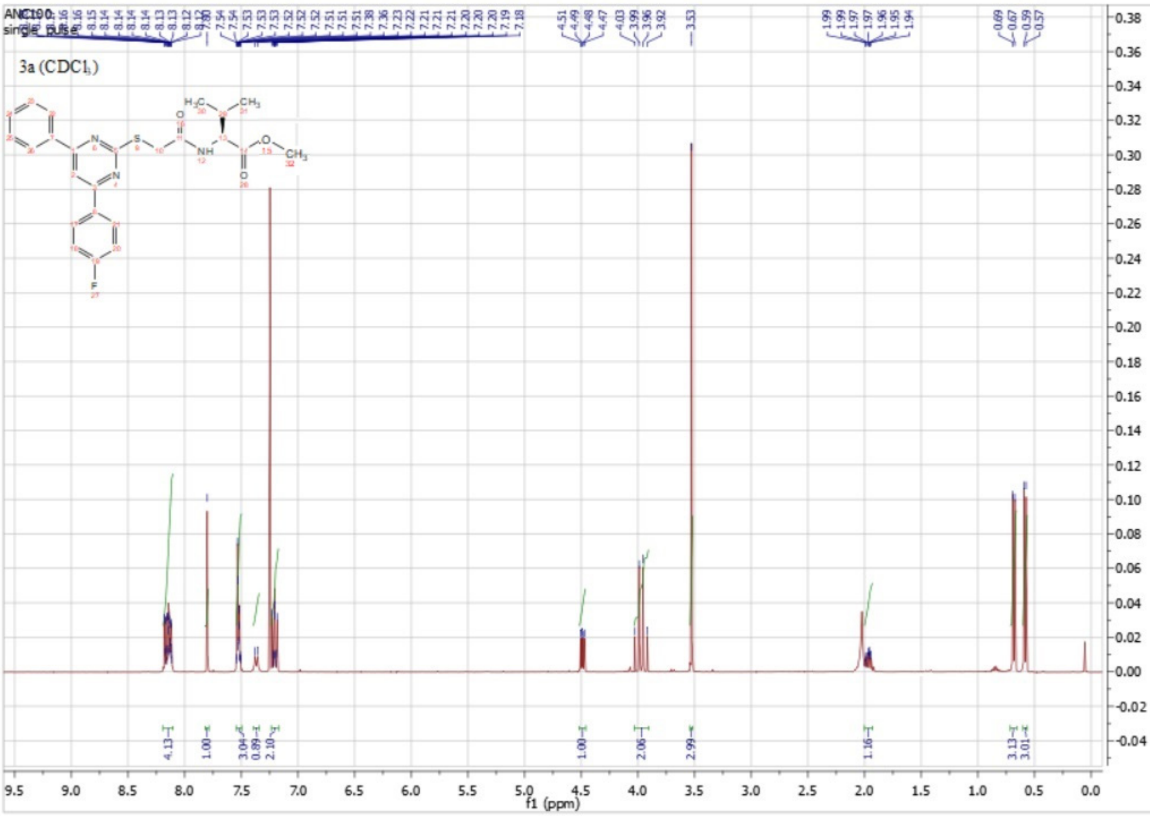
ANC100
single pulse
3a (CDCl3)
f1 (ppm)

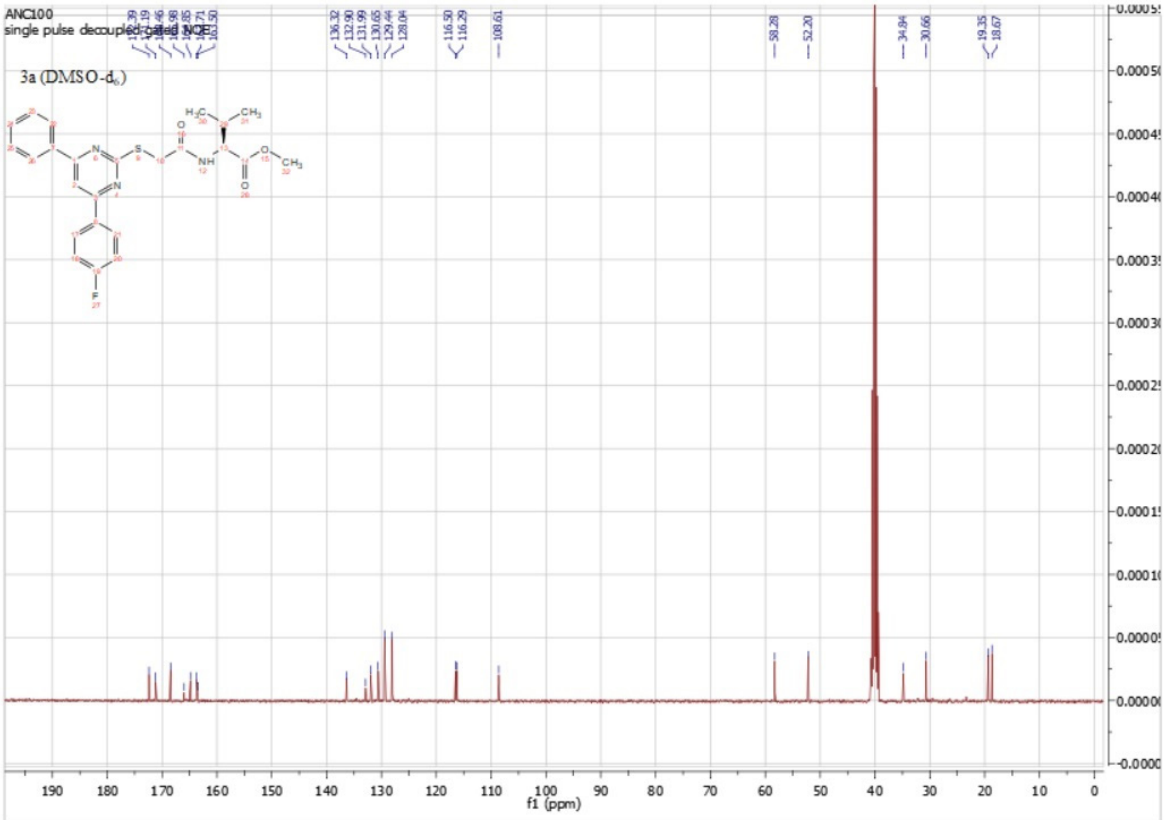
ANC100
single pulse decoupled gated NOE
3a (DMSO-d6)
f1 (ppm)

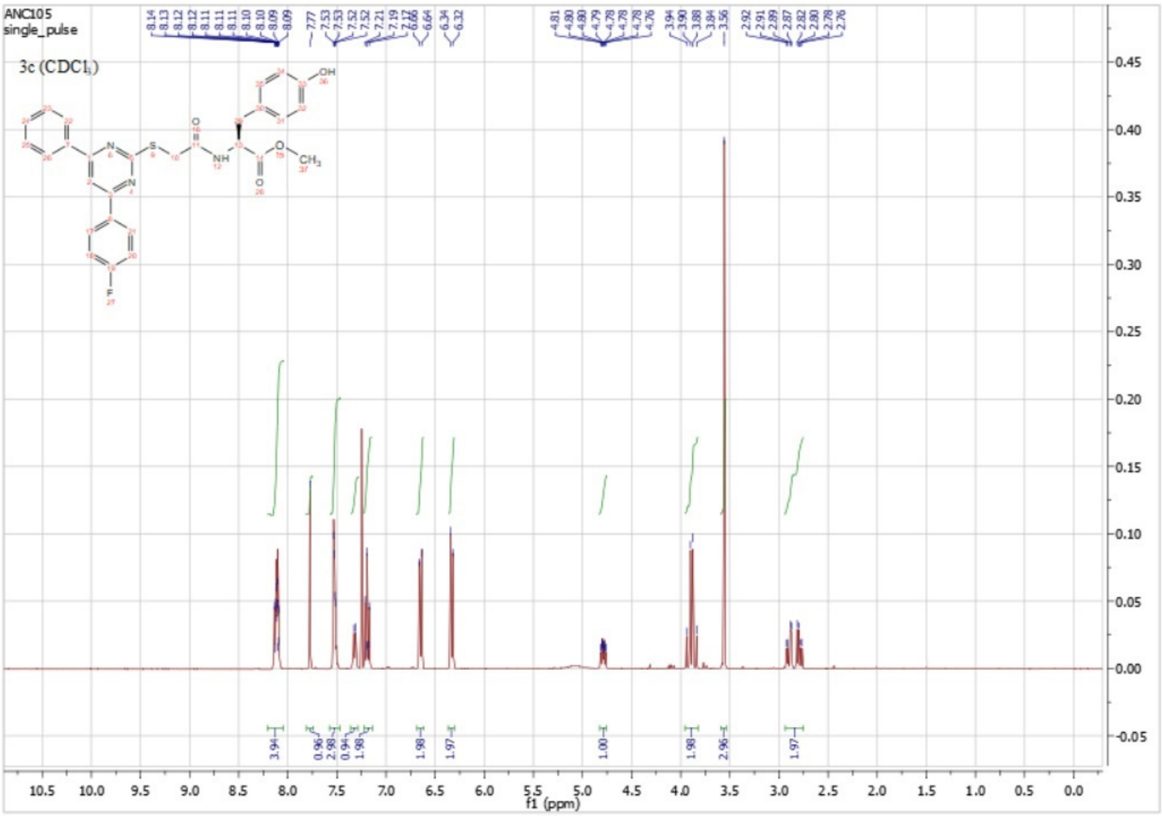
ANC105
single_pulse
3c (CDCl3)
f1 (ppm)

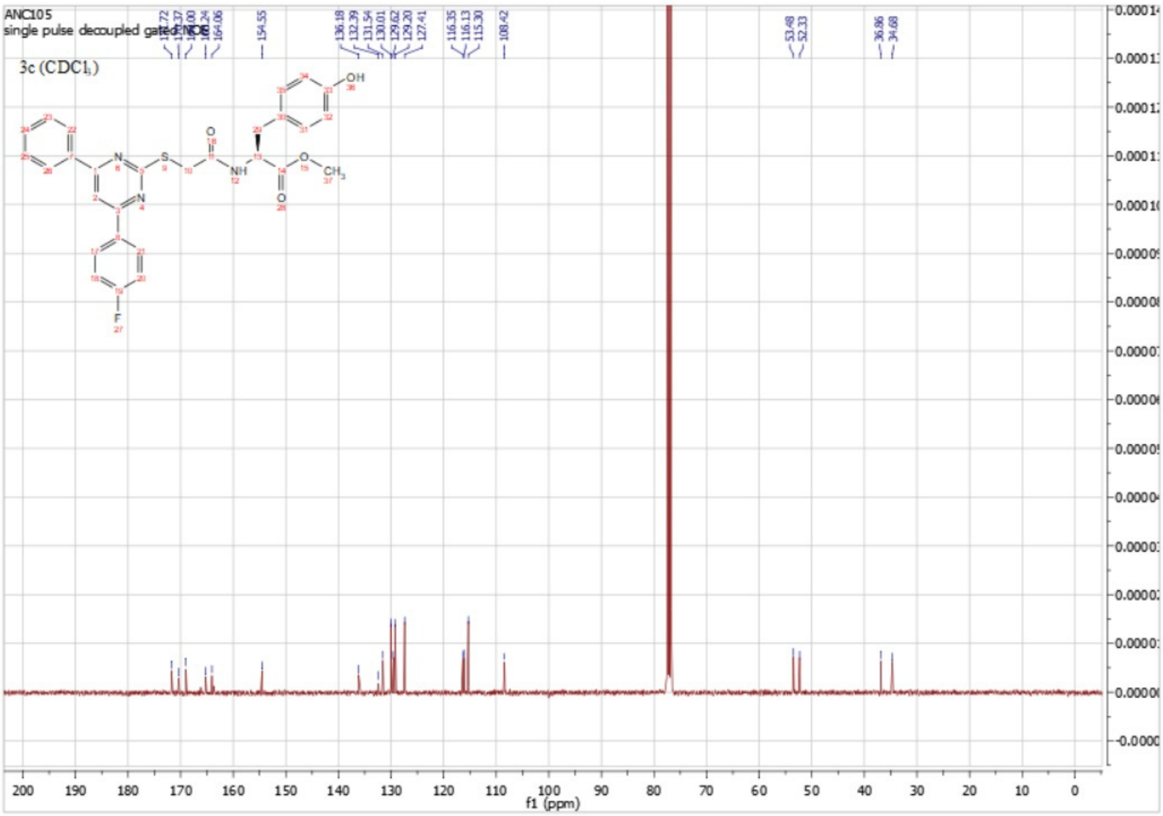
ANC105
single pulse decoupled gated NOE
3c (CDCl3)
f1 (ppm)

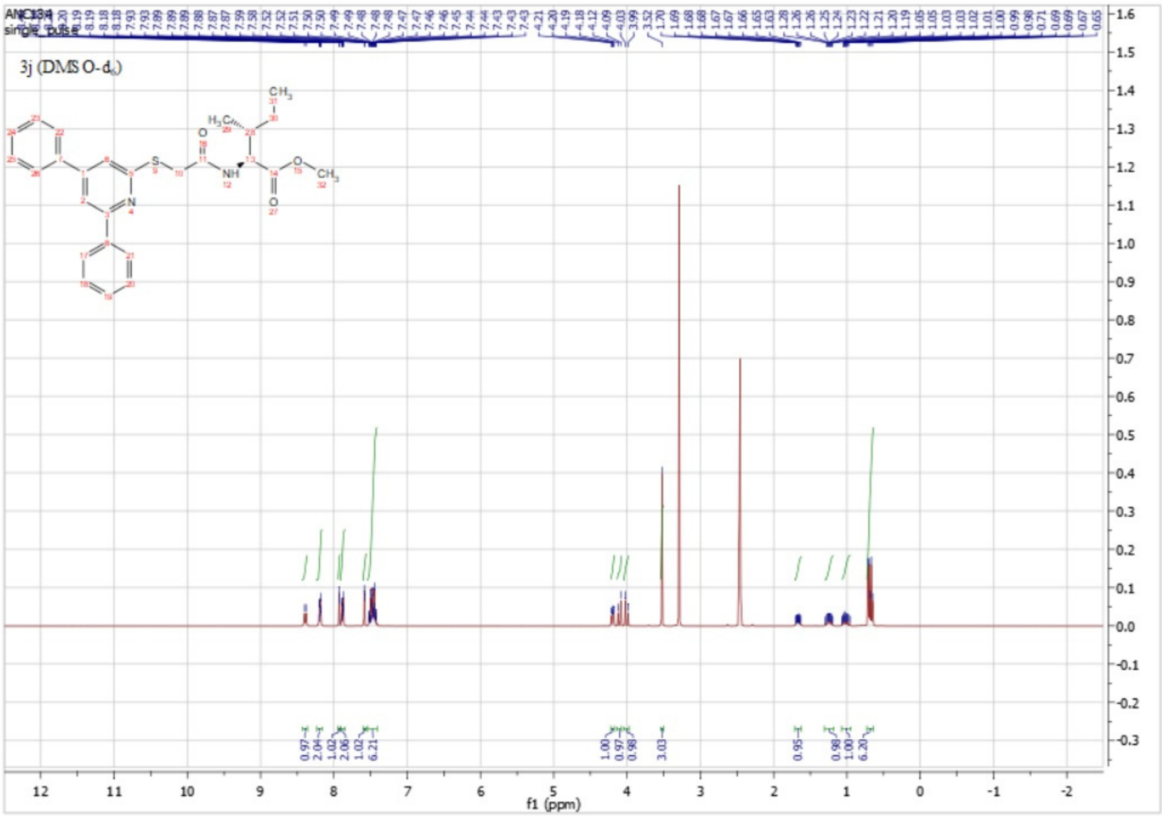
ANC134
single_pulse
3j (DMSO-d6)
f1 (ppm)

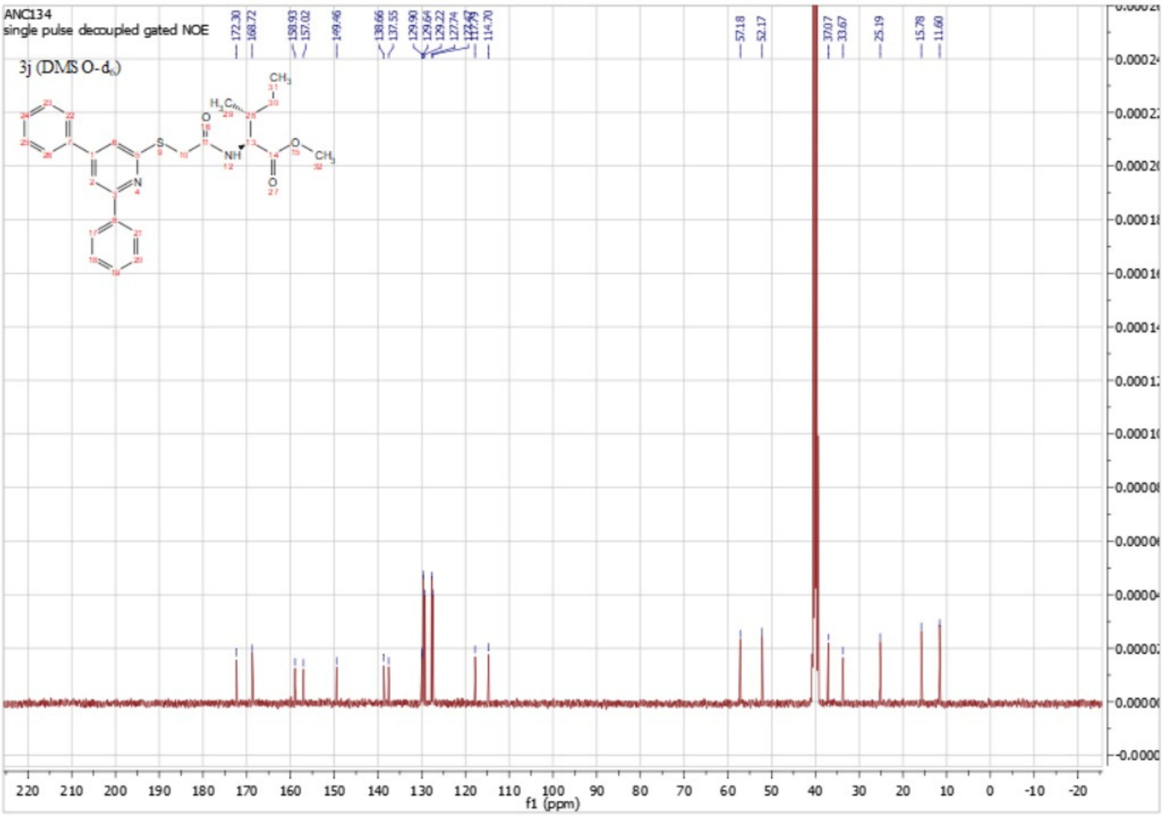
ANC134
single pulse decoupled gated NOE
3j (DMSO-d6)
172.30
168.72
158.93
157.02
149.46
138.66
137.55
129.90
129.64
129.22
127.74
57.18
52.17
37.07
33.67
25.19
15.78
11.60
f1 (ppm)

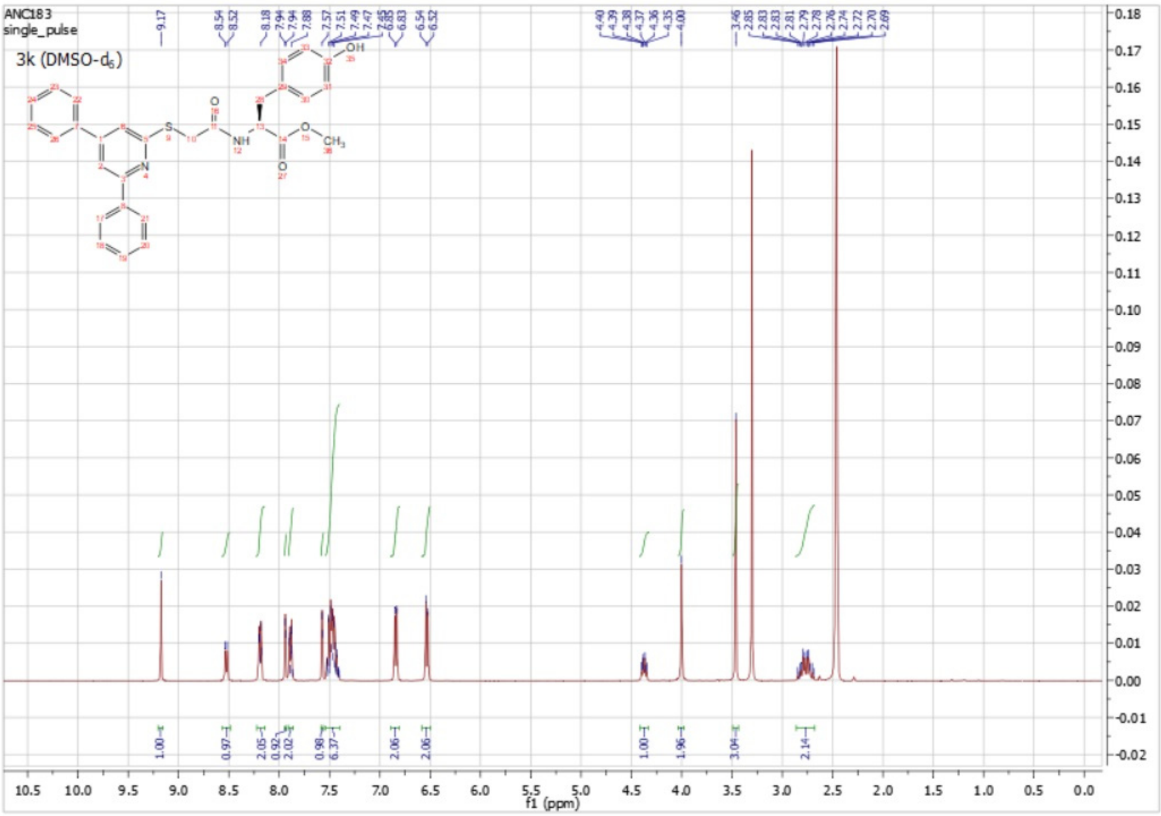
ANC183
single_pulse
3k (DMSO-d$_6$)
f1 (ppm)

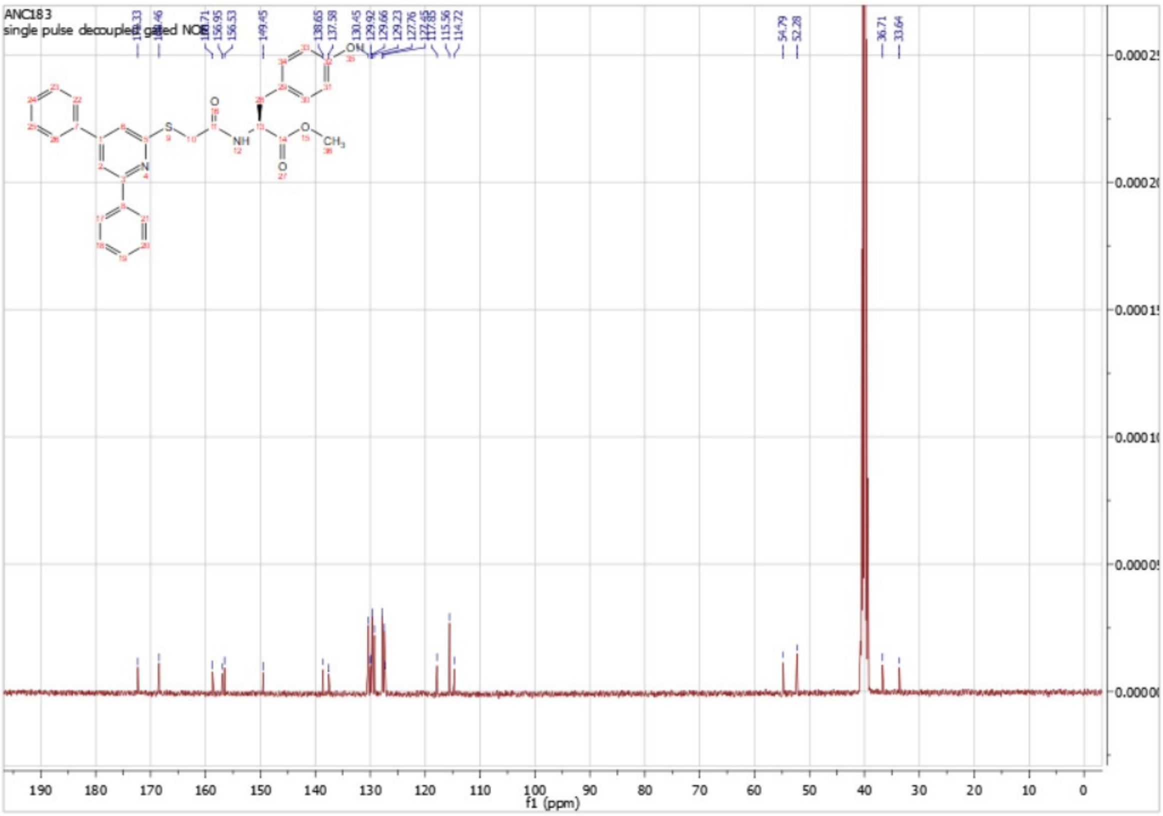
ANC183
single pulse decoupled gated NOE
f1 (ppm)
